# Proton‐Detected Solid‐State NMR of the Cell‐Free Synthesized α‐Helical Transmembrane Protein NS4B from Hepatitis C Virus

**DOI:** 10.1002/cbic.201900765

**Published:** 2020-02-20

**Authors:** Vlastimil Jirasko, Nils‐Alexander Lakomek, Susanne Penzel, Marie‐Laure Fogeron, Ralf Bartenschlager, Beat H. Meier, Anja Böckmann

**Affiliations:** ^1^ ETH Zürich Physical Chemistry Vladimir-Prelog Weg 2 8093 Zürich Switzerland; ^2^ Institut de Biologie et Chimie des Proteines MMSB Labex Ecofect UMR 5086 CNRS Université de Lyon 7 passage du Vercors 69367 Lyon France; ^3^ Department of Infectious Diseases Molecular Virology Heidelberg University Im Neuenheimer Feld 345 69120 Heidelberg Germany; ^4^ Division of Virus-Associated Carcinogenesis (Germany) Cancer Research Center (DKFZ) Im Neuenheimer Feld 242 69120 Heidelberg Germany

**Keywords:** cell-free protein synthesis, lipid reconstitution, proton detection, solid-state NMR, transmembrane proteins

## Abstract

Proton‐detected 100 kHz magic‐angle‐spinning (MAS) solid‐state NMR is an emerging analysis method for proteins with only hundreds of microgram quantities, and thus allows structural investigation of eukaryotic membrane proteins. This is the case for the cell‐free synthesized hepatitis C virus (HCV) nonstructural membrane protein 4B (NS4B). We demonstrate NS4B sample optimization using fast reconstitution schemes that enable lipid‐environment screening directly by NMR. 2D spectra and relaxation properties guide the choice of the best sample preparation to record 2D ^1^H‐detected ^1^H,^15^N and 3D ^1^H,^13^C,^15^N correlation experiments with linewidths and sensitivity suitable to initiate sequential assignments. Amino‐acid‐selectively labeled NS4B can be readily obtained using cell‐free synthesis, opening the door to combinatorial labeling approaches which should enable structural studies.

## Graphical abstract

**Membrane proteins revealed**: We show how proton‐detected 100 kHz magic‐angle‐spinning solid‐state NMR can be harnessed for investigation of eukaryotic membrane proteins in lipids. Direct NMR screening of lipid environment on sub‐milligram amounts of protein guide optimal sample preparation. Amino‐acid‐selectively labeled protein from cell‐free synthesis opens the way to structural studies.

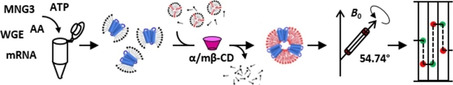

## Introduction

Membrane proteins in lipids can be studied by magic‐angle‐spinning (MAS) solid‐state NMR^1^ which, different from solution NMR, does not face overall tumbling limitations. Thus the molecular weight of biomolecular complexes that can be addressed is solely limited by spectral resolution and sensitivity. Still, analysis faced a major obstacle: complex membrane proteins often need dedicated expression systems to functionally fold, but which may deliver low yields only and are thus often incompatible with the high sample quantities (>10 mg) needed for classical ^13^C‐detected NMR approaches. This restricted NMR studies mostly to well‐expressed model membrane proteins, such as rhodopsin,1a, 1c, 1j, 2 a truncated variant of influenza A M2 channel,1e, 1f KcsA1d, 1k or BmrA.^3^ Applications of the more sensitive ^1^H‐detected MAS NMR approach to membrane proteins emerged using extensively deuterated proteins and 10–20 kHz MAS frequency.^4^ Only recently, MAS frequencies around 100 kHz have allowed to study fully proton back‐exchanged or even fully protonated proteins.^5^ The increase in MAS frequency resulted in a concomitant decrease in sample amount, to below the milligram,^6^ which presents a reduction in protein of about a factor 100 compared to ^13^C detection, that is roughly compensated by the sensitivity gain inherent to ^1^H‐detection techniques.^7^ The first 60 to 100 kHz MAS schemes have been applied to well‐established membrane protein systems,^8^ such as proteorhodopsin,^9^ outer membrane beta‐barrels,1m, 10, 11 VDAC,^11^, ^12^ a truncated variant of influenza A M2 channel,^13^ KcsA^14^ or BamA.^15^ Sample preparation of these proteins mainly followed previously established protocols using ^13^C detection, and included formation of 2D crystals1m, 12, 13 or reconstitution into liposomes at low lipid‐to‐protein ratio (LPR, *w*/*w*) by dialysis.^9^, ^15^ Such conditions are however not generally applicable to membrane proteins, and achieving optimal membrane reconstitution remains a critical step.

With sample amounts decreasing below the milligram, the use of eukaryotic protein expression systems becomes feasible for NMR sample preparation. In this context, the wheat‐germ (WG) cell‐free protein synthesis (CFPS) is a promising approach, since it provides one of the highest yields amongst eukaryotic CFPS systems, reaching routinely milligram amounts.^16^ WG‐CFPS thus presents an efficient alternative to cellular eukaryotic expression systems, and has the advantage that various NMR isotope labeling schemes, including amino‐acid selective labeling, can be easily implemented.16d, 17 Importantly, also deuteration in combination with complete amide protonation can be achieved directly during synthesis,^18^ avoiding a denaturation and refolding step, which can compromise the native fold of a membrane protein.

We here investigated the nonstructural protein 4B (NS4B) of the hepatitis C virus (HCV). Around 70 million people are chronically infected with HCV and have a high risk to develop severe liver disease, including hepatocellular carcinoma. NS4B is a transmembrane protein that is essential for HCV genome replication and virion assembly.^19^, ^20^ It has a sequence length of 261 amino acid (aa) residues (apparent molecular weight 27 kDa) and is an oligomeric α‐helical transmembrane protein constituted of three subdomains.^21^ The central subdomain contains four predicted transmembrane segments.^22^ The N‐ and C‐terminal subdomains each comprise two putative α‐helices, presumably lying on the membrane surface. Current structural information is limited to the two isolated amphipathic helices located at the N terminus, AH1 (aa 4–32, PDB ID: 2LVG) and AH2 (aa 42–66, 2JXF) as well as to the C‐terminal helix H2 (aa 229–253, 2KDR) which were all investigated by solution‐state NMR on synthetic peptides representing the described helices.^22^, ^23^ Overall, structural and topological information on full‐length NS4B remains however sparse.^24^

As NS4B is difficult to express in large quantities using conventional systems such as *Escherichia coli*, we established WG‐CFPS for NS4B in a detergent‐solubilized form.^25^ The protein can then be reconstituted into liposomes using Bio‐Bead‐enhanced dialysis.^26^ This is however a time‐consuming step and simplifying this process is thus key to enable further sample optimization.

Here, we show how lipid reconstitution of NS4B can be optimized thanks to fast lipid‐insertion schemes combined with direct screening using 2D ^1^H‐detected MAS‐NMR spectra. We show that the achieved line‐narrowing results from a decrease in inhomogeneous rather than homogeneous linewidth. Under the optimized conditions, 2D and 3D ^1^H‐detected correlation spectra can be recorded, both on fully and selectively labeled NS4B, initiating the crucial step of NMR sequential backbone assignments.

## Results and Discussion

### Cyclodextrin‐mediated reconstitution yields NS4B proteoliposomes

**Figure 1:**
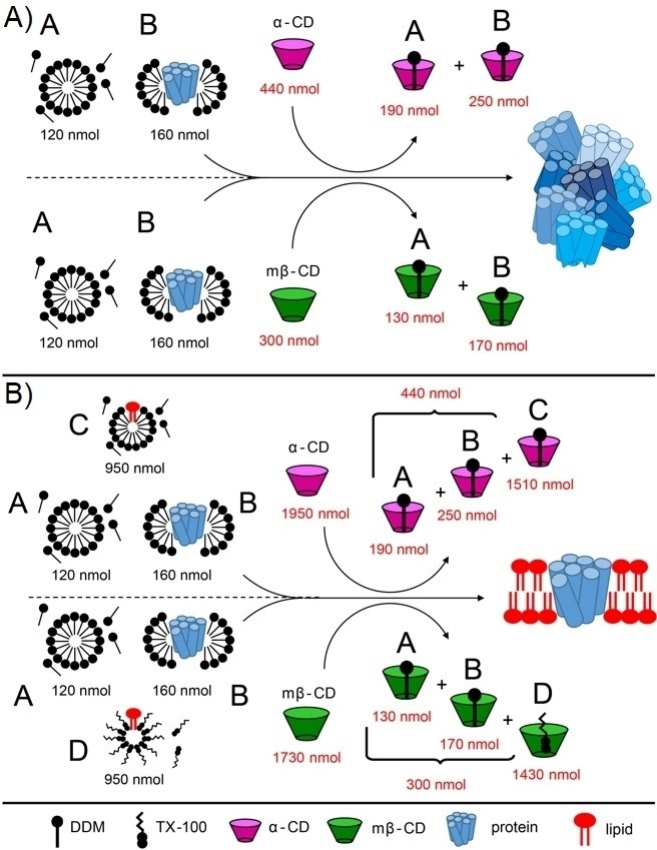
Cyclodextrin mediated lipid reconstitution of NS4B. Schematic representation of detergent removal by cyclodextrin from a 1 nmol solution of NS4B in 0.1 % DDM buffer, A) in absence, and B) in presence of detergent‐solubilized lipids. “A”, fraction of unbound DDM in a buffer; “B” fraction of DDM bound to protein; “C” and “D” DDM and Triton X‐100 respectively used for lipid solubilization. Quantities are given in black and red for detergent and cyclodextrin, respectively (for raw data see Figure S1 and for calculations see Table S1). The detergent‐to‐lipid ratio in fractions “C” and “D” is 10, and the lipid‐to‐protein ratio is 2.

Previously, we have shown that NS4B can be expressed in a soluble form using WG‐CFPS25a and that further reconstitution into liposomes allowed to record quite well‐resolved ^13^C‐detected solid‐state NMR spectra.25b Resolution in spectra using the more sensitive ^1^H detection remained however limited.25b Efficient sample optimization was hampered by the lengthy dialysis step for lipid reconstitution and complete detergent removal.3a In previous work, we adapted a faster method consisting of a combined detergent removal using cyclodextrin, as initially proposed by DeGrip et al.,27a and proteoliposome separation on a sucrose gradient,27b, 27c which speeded up the procedure by an order of magnitude. As in this approach parameters have to be fine‐tuned to avoid protein loss,27b we here use a simplified approach (Figure 1 B) consisting of gradual addition of cyclodextrin to the detergent solubilized protein and lipids, followed by gradient centrifugation. This separation into two steps is indeed easier to handle and minimizes protein loss.

In order to assess the correct amount of cyclodextrin (CD) for protein reconstitution, we first determined the minimal amount of CD necessary to bind and remove detergent molecules from the *n*‐dodecyl β‐d‐maltoside (DDM)‐solubilized NS4B micelle complexes in the absence of lipids (Figure S1 a,c in the Supporting Information),27b, 27c as outlined in Figure 1 A. Full precipitation of the protein was used as a read out for successful detergent removal. We tested two different cyclodextrins, α‐cyclodextrin (α‐CD) and methyl‐β‐cyclodextrin (mβ‐CD), and found that a total of 110 nmol of α‐CD fully precipitated 0.25 nmol of NS4B 0.1 % DDM (Figure S1 a), corresponding to 440 nmol per 1 nmol of NS4B (Figure 1 A, upper panel). In case of mβ‐CD, 75 nmol was used up per 0.25 nmol of NS4B (Figure S1 c), resulting in a total of 300 nmol per 1 nmol of NS4B (Figure 1  A lower panel).

In a second step, we estimated the amount of CD necessary to remove additional DDM or Triton X‐100 detergents used for lipid solubilization. Experimental details of the precipitation assay in the presence of solubilized lipids or additional Triton X‐100 are summarized in Figure S1 b and c, as well as in the Supporting results and Table S1. As a result (Figure 1 B), 1950 nmol of α‐CD is needed to reconstitute 1 nmol of NS4B into DDM‐solubilized lipids, while 1730 nmol of mβ‐CD is required for reconstitution into Triton X‐100‐solubilized lipids. From these experiments, the number of NS4B‐bound DDM molecules was estimated as 160 (Supporting results and Table S1), in agreement with previously published data, where micelles with model membrane proteins contain around 150–300 DDM monomers.^28^ Further, we determine that α‐CD binds at an approximate ratio of 1.6 to DDM, mβ‐CD at 1.1 to DDM and mβ‐CD at 1.5 to Triton X‐100 (Supporting results and Table S1), which correlates with previous experiments showing that cyclodextrin molecules do not necessarily bind detergents in 1:1 ratio.27a, 29

We then prepared proteoliposomes using the above‐described protocol and cyclodextrin amounts. The reconstituted protein was analyzed on a sucrose gradient, and a visible opaque band in the gradient was detected, which correlated with the expected protein bands of NS4B on an SDS PAGE gel (Figure S1 d, e). No further signals were detected by SDS PAGE, indicating that virtually all protein formed proteoliposomes. The protocol established here allowed to gain a factor of 10 in time, which also makes the reconstitution step more robust with respect to protein degradation.

After this biochemical assessment, we directly optimized the amount and type of cyclodextrin by NMR. To enhance readability of the spectra, we used a selectively Gly‐ and Tyr‐labeled NS4B sample (dGY NS4B). We reconstituted NS4B into PC/Chol (phosphatidylcholine/cholesterol) at LPR 2 using different conditions, as detailed in Figure S2 and Table S2. As a result, the 2.5‐fold excess of mβ‐CD over the minimal amount determined, combined with PC/Chol solubilized in Triton X‐100, yielded the spectra with best resolution and signal‐to‐noise ratio (SNR), and this condition was thus set as the sample preparation standard. Finally, 2D ^1^H,^15^N correlation spectra (Figure S3) confirmed that NS4B lipid reconstitution using cyclodextrin indeed yielded very similar NMR spectra as the more time‐consuming reconstitution using Bio‐Bead‐enhanced dialysis,25b and could thus be used as starting point for further optimizations.

### Proton linewidths depend on the lipid environment

**Figure 2:**
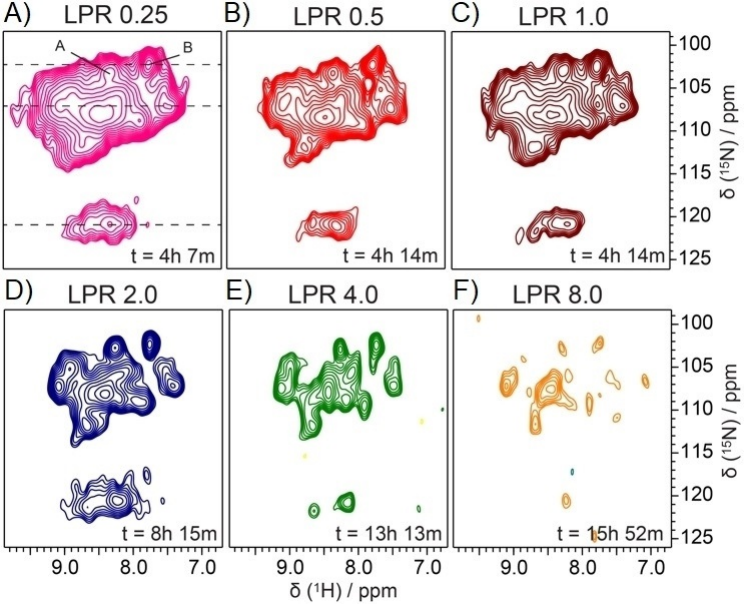
Spectral resolution of 2D ^1^H,^15^N spectra as a function of LPR. Two‐dimensional ^1^H,^15^N correlation spectra of dGY NS4B reconstituted into PC/Chol liposomes at LPRs from 0.25 to 8. Two‐dimensional ^1^H,^15^N correlation spectra were acquired at 850 MHz, 80 kHz (A–D) or 90 kHz (E, F) MAS and 23 °C. Dashed lines represent the position of 1D traces shown in Figure S4 Lipid reconstitution was achieved by Bio‐Bead‐enhanced dialysis (A–C) or by using mβ‐CD (D–F). Full width at half maximum (FWHM) and SNR are given in Table S3.

Next, we compared spectra recorded at different LPRs to identify optimal conditions. For maximal protein NMR signal, the LPR, in principle, should be as low as possible. On the other hand, a sufficient amount of lipids is important to ensure that the protein is well folded.^30^ We therefore compared dGY NS4B reconstituted into PC/Chol lipids at various LPRs. The resulting 2D ^1^H,^15^N correlation spectra (Figure 2) reveal that the spectral linewidth improves by about a factor of two from LPR 0.25 to LPR 4 (Figure S4, Table S3). Increase of LPR requires an increase in measurement time, which becomes prohibitive at LPRs exceeding about 2. We therefore chose LPR 2 as the best compromise between a narrow spectral linewidth and high SNR (Figures 2 and S4, Table S3).

**Figure 3:**
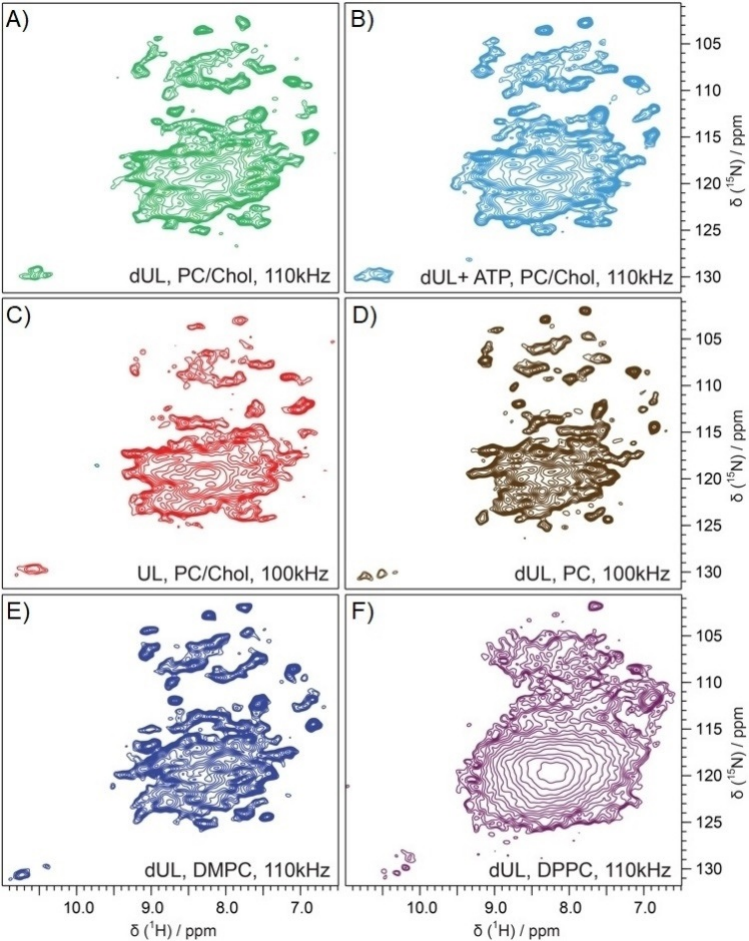
^1^H‐detected 2D ^1^H,^15^N spectra as a function of the composition of the lipid environment. A, B, D, E, F) dUL NS4B, or C) UL NS4B reconstituted in liposomes (LPR 2) with different lipid composition: A)–C) PC/Chol, D) PC, E) DMPC and F) DPPC. B) NS4B was reconstituted into lipids in the presence of ATP. Spectra were recorded at 850 MHz and MAS frequency of 110 kHz. All spectra were acquired at 23 °C.

Not only LPR but also lipid membrane thickness, hydrophobic mismatch, the presence of cholesterol and the bilayer fluidity, among others, can influence protein folding and the dynamic properties of the protein in the membrane.^31^ We thus investigated the dependence of NMR spectral parameters on selected lipids. For this, [^2^H,^15^N,^13^C] (dUL) NS4B as well as [^1^H,^15^N,^13^C] (UL) NS4B was reconstituted at LPR 2 into different lipids as detailed in Figure 3.

To obtain a proxy for the spectral quality of NS4B in the different lipid environments, we determined the average ^1^H FWHM and SNR for 9–10 isolated peaks (Table 1). A comparison (Figure 3 and Table 1) of linewidths showed that NS4B reconstituted into PC, both in the presence and absence of 30 % cholesterol, as well as NS4B reconstituted into DMPC lipids, gave spectra with an average spectral linewidth between 100–120 Hz for isolated peaks. In contrast, NS4B reconstituted into DPPC lipids showed only poorly resolved spectra.

**Table 1:** Homogeneous and inhomogeneous ^1^H and ^15^N linewidths of NS4B in different lipid environments.

					^1^H					^15^N	
						Δ^total^	Δ^homo^	Δ^inhomo^			
Labeling	Lipids	*T*_m_^[a]^	hNH^[b]^	MAS^[c]^	SNR^[d]^	FWHM^[e]^	*R*_2_′/*π* ^[f]^	FWHM−*R*_2_′/*π* ^[g]^	*R*_1ρ_/*π* ^[h]^	*R*_2_′/*π* ^[i]^	*R*_1ρ_/*π* ^[j]^
		[°C]	Figure 3	[kHz]		[Hz]	[Hz]	[Hz]	[Hz]	[Hz]	[Hz]
dUL	PC/Chol		A	110	7±2	120±20	60±10	60±30	29±1	14±1	3±1
dUL/ATP	PC/Chol		B	110	9±3	120±30	60±10	60±30	30±1	15±1	3±1
UL	PC/Chol		C	110	4.5±1.2	140±30	150±10	0±30	174±6	12±1	3±1
dUL	PC	‐6	D	100	8±2	100±20	70±10	30±30	34±2	18±1	11±1
dUL	DMPC	24	E	110	11±3	110±30	60±10	50±30	36±1	15±1	5±1
dUL	DPPC	41	F	110	–	–	80±10	–	26±1	24±1	3±1

[a] “*T*_m_”, lipid phase‐transition temperature; [b] “hNH”, panel in Figure 3 with the respective spectrum; [c] “MAS”, spinning frequency; [d] “SNR”, average SNR for 9 or 10 isolated peaks (for individual values see Table S5); [e] “Δ^total^”, average ^1^H FWHM of the same peaks; [f] “Δ^homo^”, bulk ^1^H homogeneous linewidth as measured in a Hahn‐echo *t*_2_′ experiment (Figure S7); [g] “Δ^inhomo^”, bulk ^1^H inhomogeneous linewidth calculated from Δ^total^−Δ^homo^; [h] “*R*_1ρ_/*π*”, proton rotating‐frame relaxation time extracted from a bulk ^1^H *R*_1ρ_ experiment (Figure S7); [i] ^15^N “*R*_2_′/*π*” and [j] ^15^N “*R*_1ρ_/*π*”, ^15^N bulk relaxation‐rate constants calculated by taking the inverse of *T*_2_′ and *T*_1ρ_ times that were fitted from intensity decay curves (Figure S7).

In solid‐state NMR, the total linewidth (Δ^total^) has a homogenous (Δ^homo^) and inhomogeneous (Δ^inhomo^) contribution (Δ^total^=Δ^homo^+Δ^inhomo^). The homogeneous part represents coherent effects (Δ^coherent^) caused by incomplete averaging of the dipolar interaction, and incoherent effects (Δ^incoherent^) driven by molecular dynamics, and thus Δ^homo^=Δ^coherent^+Δ^incoherent^. The inhomogeneous part reflects sample and field inhomogeneity.^32^ To determine the homogenous contribution (Δ^homo^=*R*_2_′/*π*) to the total linewidth we measured bulk ^1^H R_2_′ relaxation‐rate constants of amide groups using a Hahn‐echo experiment.^33^ As Δ^coherent^ depends mainly on the geometry of the proton spin system,^32^ it should be similar in comparable secondary structure elements. In rigid, α‐helical parts of deuterated (and HN back‐exchanged) proteins, typical values of Δ^homo^ are around 30 Hz at 100 kHz MAS in the absence of dynamics (Δ^incoherent^≃0).^34^ For dUL NS4B, we found values around 60–80 Hz (Table 1) suggesting that Δ^incoherent^ is about 30–50 Hz, which indicates that the linewidth may be influenced by dynamics. This value derived for Δ^incoherent^ is similar to the measured ^1^H R_1ρ_ transverse relaxation rate constant (measured at a spinlock field of 13 kHz and tabulated in Table 1 as the rate constant divided by *π* and thus representing a linewidth). Δ^total^ is broadest for the protonated systems, but the differences are small, which might be due to the significant experimental error bars. We also measured ^15^N *R*_1ρ_ and *R*_2_′ rate constants (Table 1), which are, as expected, much lower than for protons. Interestingly, a comparison of Δ^homo^ of DUL NS4B showed only small differences for the different lipid environments, including in the badly resolved spectra of NS4B in DPPC lipids (Table 1). Therefore, the difference in total linewidth between NS4B in DPPC (Figure 3 F) and in the other lipids (Figure 3 A, D, E) must be predominantly due to inhomogeneous line broadening and might be correlated to the different phase‐transition temperatures of the various lipids (Table 1, see also discussion below).

To assess whether the observed linewidth is related to the lipid phase‐transition temperature (*T*_m_), we recorded spectra of NS4B in different lipid environments and at different temperatures (Figure S5). While for NS4B in PC liposomes, which show a *T*_m_ of −6 °C,^35^ the temperature dependence seems only weak (Figure S5 A,C), for DMPC, with a *T*_m_ of 24 °C, the spectral resolution clearly decreases from 21 to −6 °C (Figure S5 B, D). This suggests that spectral resolution is indeed influenced by the experimental temperature relative to T_m_. This is similar to previous findings for the β‐barrel membrane protein OmpX in lipid nanodiscs.10b A comparison of Δ^homo^ and also the ^1^H *R*_1ρ_/*π* rate constants indicate only very little difference between the two temperatures, independent of the lipid environment (Table S4). Thus, in conclusion, the difference in spectral resolution cannot be explained by dynamics‐related homogenous line broadening alone (Table S4), but inhomogeneous line broadening below the lipid phase transition temperature has an important contribution as well.

We also recorded the spectra of NS4B prepared in the presence of ATP. NS4B is able to hydrolyze ATP,^36^ which might be essential for NS4B function in the HCV life cycle.^37^ We thus speculated that the addition of ATP during the reconstitution step could affect NS4B folding and improve sample quality. Chemical shifts and linewidths in ^1^H,^15^N correlation spectrum of NS4B‐ATP sample were however comparable to the sample prepared in the absence of ATP (Figure 3 B). At the same time, approximately 20 % improvement of SNR as a direct or indirect effect of ATP was observed, which resulted in selection of this sample for 3D experiments (see below).

### 3D experiments on fully and selectively labeled NS4B for sequential backbone assignments

A set of ^1^H‐detected 3D experiments at 110 kHz MAS was acquired to evaluate the suitability of the dUL NS4B sample for sequential backbone assignment, a crucial step for further structural NMR analysis of the protein. Three spectra, hCANH, hCONH and hCAcoNH,6a, 34, 38 were recorded at 110 kHz MAS and an hCBcaNH spectrum^34^, ^39^ was acquired at 60 kHz MAS frequency in a 1.3 mm rotor (Table S6).

Out of 248 expected resonances, prolines and flexible C‐terminal tag not counted, approximately 190, 155 and 100 resonances were picked by the automatic peak‐picking routine of the CCPN software package in the hCANH, hCONH and hCAcoNH spectra, respectively. Out of the 27 expected Gly residues, if nine residues in the flexible tag are neglected, 22 resonances in the Gly spectral region were visible in the hCANH spectrum. Although 3D spectra still show signal overlap, more than 60 peaks in the hCANH spectrum could be connected to their counterparts in the hCONH spectrum, as shown for a number of examples in Figure S6.

**Figure 4:**
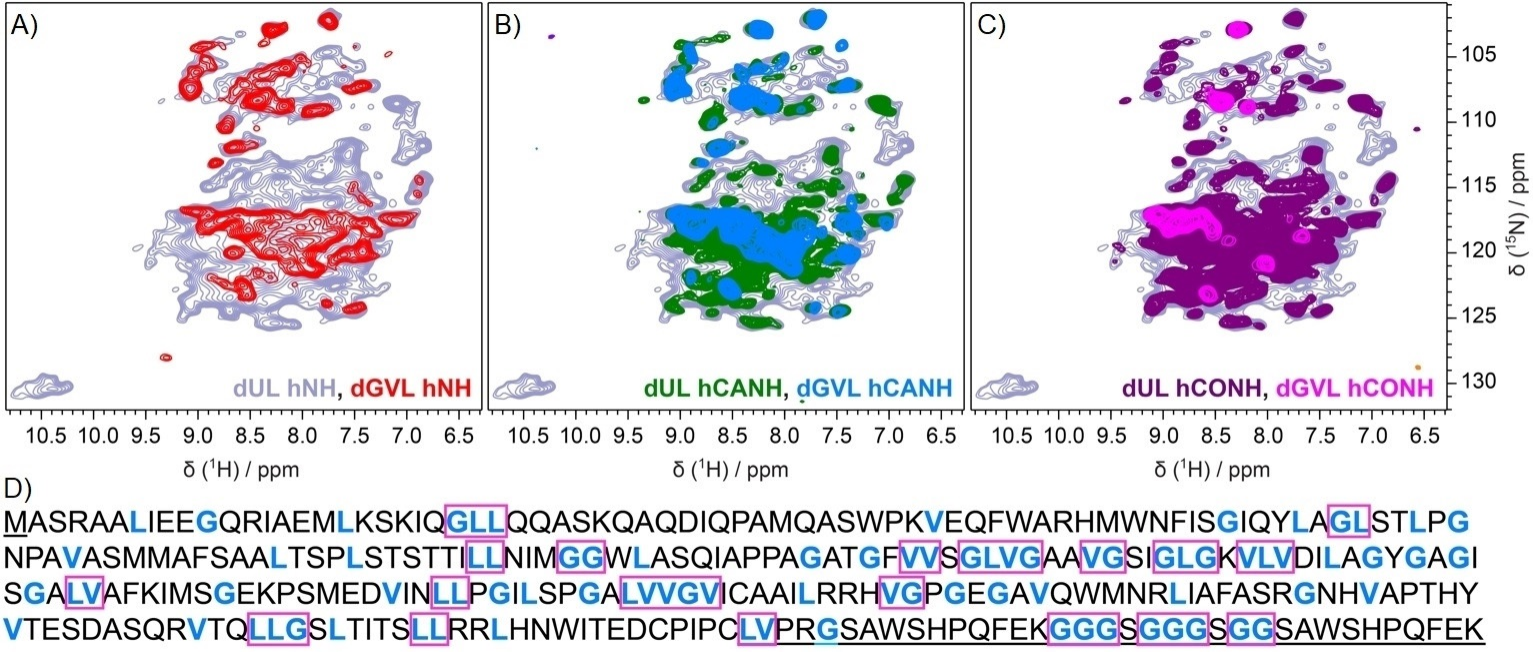
Selectively labeled dGVL NS4B. [^1^H,^15^N] 2D correlation spectra of dUL NS4B reconstituted at LPR 2 with ATP (in grey) overlaid with A) the dGVL NS4B [^1^H,^15^N] 2D correlation spectrum in red, B) the projection of the 3D hCANH spectra of dUL NS4B in green and dGVL NS4B in blue, and C) the projection of the 3D hCONH spectra of dUL NS4B in violet and dGVL NS4B in magenta. D) NS4B sequence with Gly, Val and Leu amino acids highlighted. Consecutive pairs that result in expected resonances in the 3D hCONH spectrum are shown in magenta boxes. The N‐terminal methionine (attached for translation initiation) as well as the C‐terminal thrombin cleavage site, GSA linker and tandem *Strep*‐tag II are underlined.

To reduce the significant signal overlap in central regions of the spectra, the recording of 4D spectra^40^ would be useful. For SNR reasons we instead turned to selectively labeled samples to decongest the spectra and obtain anchor points for the sequential backbone assignments. We prepared a deuterated Gly, Val and Leu selectively labeled NS4B sample (dGVL NS4B). Three‐dimensional hCANH and hCONH spectra were recorded and their ^13^C projections are shown on the ^1^H,^15^N dUL NS4B planes in Figure 4 B and C, respectively. Although, in principle, all labeled residues in the sequence will contribute resonances in the hCANH spectrum, for the hCONH spectrum only pairs of either Gly, Val and Leu will contribute to a resonance signal, as both the C′(*i*−1) and the N(*i*) have to be isotopically labeled.

The automated peak picking selected 44 peaks in the hCANH spectrum, out of 74 expected resonances. Eleven resonances were picked in an hCONH spectrum out of expected 24 intraresidue correlations. Eight out of 11 correlations in the dGVL NS4B hCONH spectrum could be connected to residues in the hCANH dUL NS4B spectrum. Out of those, two Gly and one Leu could be assigned, namely L123, G125 and G238. One signal in the hCONH spectrum was assigned to a double Gly motif, which can be found once in the NS4B sequence and five times in the twin strep tag sequence. It is likely that this single GG pair belongs to NS4B rather than the tag, which is presumably flexible and therefore invisible in a CP‐based experiment.

**Figure 5:**
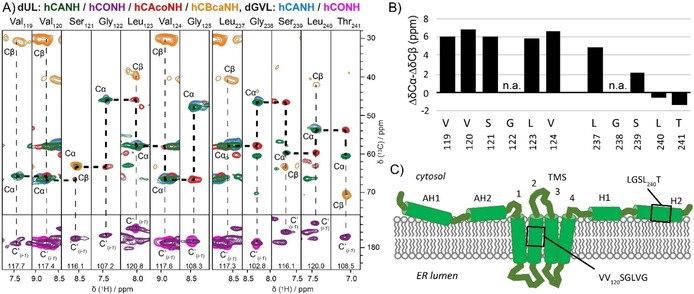
3D correlation spectra of deuterated NS4B allow to establish sequential connectivities. A) Selected strip plots representing the assignment of amino‐acid stretches VV_120_SGLVG and LGSL_240_T using 3D hCANH (green), hCONH (violet) and hCAcoNH (red) spectra recorded on dUL NS4B, and hCANH (cyan) and hCONH (magenta) of dGVL NS4B in PC/Chol lipids LPR 2, at 110 kHz MAS. The dUL NS4B hCBcaNH spectrum (orange) was acquired at 60 kHz MAS. B) Secondary chemical shift differences, Δ*δ*_Cα_−Δ*δ*_Cβ_ of the two stretches from (A). C) Putative topology model of the NS4B protein adapted from Gouttenoire et al.,^22^ in which NS4B is proposed to contain eight α‐helices. N‐ and C terminus harbor four presumably amphipathic α‐helices: AH1 (aa 4–32, PDB ID: 2LVG), AH2 (aa 42–66, 2JXF), H1 (predicted between aa 201–212) and H2 (aa 229–253, 2KDR). The central part (aa 70–190) contains four predicted transmembrane segments. The black boxes indicate the location of the two assigned amino acid stretches VV^120^SGLVG and LGSL^240^T, respectively. Random‐coil chemical shifts used for the calculation of secondary chemical shifts were taken from Zhang et al.^41^.

Combining information from the 3D hCANH, hCONH, hCAcoNH and hCBcaNH spectra^34^ of uniformly and selectively labeled samples, we were able to identify two amino‐acid stretches, comprising residues from Val119 to Gly125 (VV^120^SGLVG) and from Leu237 to Thr241 (LGSL^240^T). Corresponding strip plots are shown in Figure 5. Δ*δ*_Cα_−Δ*δ*_Cβ_ secondary chemical‐shift differences of the amino‐acid stretch VV^120^SGLV gave positive values (Figure 5 B), suggesting α‐helical secondary structure, in agreement with previously proposed topological models^22^, ^36^ (Figure 5 C). On the other hand, within the amino‐acid stretch LGSL^240^T, a positive Δ*δ*_Cα_−Δ*δ*_Cβ_ secondary chemical‐shift difference for N‐terminal leucine is observed, whereas Ser, Leu and Thr show values close to 0 (Figure 5 B), suggesting no defined secondary structure for that part. This is in conflict with a topological model based on a solution‐state NMR analysis of an isolated peptide, which suggested full α‐helical character for the entire region.^22^, 23b However, our data might support a more complex picture which was proposed for full‐length NS4B, in which the C‐terminus containing a putative walker B motif has been described to interact with the loop between *trans*‐membrane helices 2 and 3, comprising a walker A motif.^36^

Further spectral backbone assignment is currently challenged by a lack in spectral resolution and sensitivity. Indeed, the high spectral overlap of the mainly α‐helical NS4B resonances, in combination with significant transverse relaxation and concomitant low signal to noise due to the membrane insertion at relatively high LPR, are problematic for less sensitive experiments, such as for example, the hCBcaNH experiment, and also experiments which allow for the identification of sequential N(*i*) and N(*i*−1) connectivities.^42^, ^43^ Although such experiments are feasible in principle, their overall low transfer efficiency prevents data collection with present‐day equipment. Currently, recording of 3D hCANH and hCONH spectra on different selectively labeled samples is therefore the most promising approach to obtain further sequential backbone assignments. Sophisticated combinatorial labeling schemes devised in the context of solution NMR studies^44^ can inspire further solid‐state NMR approaches for sequential backbone assignment employing a combination of selectively labeled samples. Still, also here SNR is an issue, and higher magnetic field strength should boost the SNR and reduce signal overlap in the near future. Even faster magic angle spinning will increase transfer efficiencies not only in CP as used here, but also in *J* coupling‐based experiments. For instance, changing from 100 kHz to 200 kHz MAS frequency will elongate the transverse *t*_2_′ times by roughly a factor of 2^32^, ^45^ and possibly overcompensate the SNR loss by the smaller sample amount in faster and therefore smaller rotors.

## Conclusion

We show for the cell‐free synthesized α‐helical integral membrane protein NS4B of HCV in membranes that solid‐state NMR spectra could be recorded in a reasonable amount of measurement time on a membrane protein sample reconstituted in lipids at LPR 2 in a 0.7 mm rotor. We screened for optimal sample preparation using rapid lipid reconstitution via cyclodextrin addition, and assessed the best lipid‐to‐protein ratio directly on ^1^H‐detected solid‐state NMR spectra. Relaxation measurements confirmed the expected narrower homogeneous linewidth of deuterated protein compared to protonated one, and revealed that inhomogeneous line broadening was substantial, and strongly dependent on the lipid chosen, which is likely related to the lipid phase transition temperature. The evaluation of different lipids showed that reasonably resolved spectra can be reproducibly recorded, and that most conditions yield similar spectra, with the notable exception of DPPC. Three‐dimensional experiments were recorded and, in principle, provide the basis for sequential backbone assignments. Still, spectral overlap is substantial, and we showed that selectively labeled samples, straightforward to obtain by CFPS, can be used to identify anchor points for sequential assignments. Further improvement in resolution is however compulsory to progress to complete backbone assignment, and with current hardware, the use of combinatorial labeling is thus the most promising approach.

## Conflict of interest

*The authors declare no conflict of interest*.

## Supporting information

As a service to our authors and readers, this journal provides supporting information supplied by the authors. Such materials are peer reviewed and may be re‐organized for online delivery, but are not copy‐edited or typeset. Technical support issues arising from supporting information (other than missing files) should be addressed to the authors.

Supplementary
