# Supplementary material for: Proton‐Detected Solid‐State NMR of the Cell‐Free Synthesized α‐Helical Transmembrane Protein NS4B from Hepatitis C Virus
Source: Chembiochem. 2020 Feb 20;21(10):1453–60. doi: 10.1002/cbic.201900765 (PMC7318649; doi:10.1002/cbic.201900765)
Supplement: Supplementary file 1 — Supplementary [file CBIC-21-1453-s001.pdf]

CHEMBIOCHEM

Supporting Information

Proton-Detected Solid-State NMR of the Cell-Free Synthesized α-Helical Transmembrane Protein NS4B from Hepatitis C Virus

Vlastimil Jirasko+,[a] Nils-Alexander Lakomek+,[a] Susanne Penzel,[a] Marie-Laure Fogeron,[b] Ralf Bartenschlager,[c, d] Beat H. Meier,*[a] and Anja Böckmann*[b]

cbic_201900765_sm_miscellaneous_information.pdf

Supporting Information

Proton-detected solid-state NMR of the cell-free synthesized α-helical transmembrane protein NS4B from hepatitis C virus

Vlastimil Jirasko[a]‡, Nils-Alexander Lakomek[a]‡, Susanne Penzel[a], Marie-Laure Fogeron[b], Ralf Bartenschlager[c], Beat H. Meier[a]* and Anja Böckmann[b]*

Table of Contents

Supporting Experimental Section 2

Plasmids 2

Wheat germ cell-free protein synthesis 2

Purification of full-length NS4B by affinity chromatography 2

Reconstitution of NS4B into proteoliposomes 3

Precipitation assay 4

Analysis of reconstituted NS4B by sucrose density-gradient ultracentrifugation 4

Preparation of dUL NS4B samples in the presence of ATP used for three-dimensional NMR experiments at 110 kHz 5

Solid-state MAS NMR experiments 5

Supporting Results 7

Supporting Figures 8

Supporting Tables 17

References 24

Supporting Experimental Section

Plasmid

The pEU-E01-NS4B-TWS plasmid encoding gene of full-length NS4B protein from HCV (strain JFH-1, genotype 2a, GenBank accession number AB047639), was described previously [1]. The recombinant protein consisted of methionine for translation initiation, the NS4B sequence followed by a thrombin cleavage site (TCS), GSA linker and a tandem of *Strep*-tag II sequences (TWS - TWin *Strep* tag). "M-NS4B-TCS-GSA-TWS" recombinant protein is referred in this work as "NS4B" protein. Transcription was started from the SP6 promotor sequence and terminated approximately 1600 bp downstream of the open reading frame stop codon. Protein expression was driven by the cap-independent, artificial E01 translation enhancer sequence. The tandem of *Strep*-tag II sequence was not cleaved of from the protein in this work. The plasmid DNA was transformed into *Escherichia coli* DH-5α chemically competent cells (Life Technologies), isolated with a NucleoBond Xtra Maxi kit (Macherey-Nagel) and purified by a phenol/chloroform extraction.

Wheat germ cell-free protein synthesis

WG-CFPS using homemade wheat germ extracts was performed as described previously [1-2]. Briefly, transcription and translation steps were carried out separately. Transcription was performed for 6 hours at 37 °C in 1x Trancription buffer (CellFree Sciences), RNAsin (1U/µl) (CellFree Sciences), SP6 polymerase (1U/µl) (CellFree Sciences), 10 mM rNTP mix (Promega) and 0.1 µg/µl of plasmid DNA. Translation was performed for 16 hours at 22°C without shaking using the bilayer method. Translation mixture adapted for 6-well plates format consists of 0.5 ml of reaction mix at the bottom overlaid by 5.5 ml of feeding buffer. The reaction mix contained 0.25 ml of transcription reaction, 0.25 ml of wheat germ extract, 40 ng/ml creatine kinase (Roche), 6 mM amino acid mixture (average concentration of each amino acid 0.3 mM) and 0.07% (w/vol) MNG-3 (Maltose Neopentyl Glycol-3, Anatrace). The feeding buffer contains 1x SubAmix buffer (CellFree Sciences), 0.1 % MNG-3 and 6 mM amino acid mixture. Unlabelled amino acids were used for small scale tests analyzed by ultracentrifugation and SDS-PAGE. NMR samples were prepared with [^{2}H,^{13}C,^{15}N] or [^{13}C,^{15}N]-labelled amino acids (Cambridge Isotope Laboratories, Inc.) in case of uniformly labelled proteins. Selectively labelled proteins dGY and dGVL NS4B were prepared by mixing of [^{2}H,^{13}C,^{15}N]-labelled Glycine and Tyrosine or Glycine, Valine and Leucine respectively with remaining 18 or 17 [^{2}H] labelled amino acids (Cambridge Isotope Laboratories, Inc.).

Purification of full-length NS4B by affinity chromatography

After 16 h, the total cell-free reaction was pooled and incubated with benzonase at room temperature on a rolling wheel for 30 min, followed by incubation with 0.25% (w/vol) DDM (N-Dodecyl-beta-Maltoside, Affymetrix) for 30 min to exchange from MNG-3 to DDM detergent. Total reaction was then centrifuged at 40,000 *g* and 4 °C for 40 min and the supernatant was loaded on a column containing *Strep*-Tactin high capacity gravity resin (IBA Lifesciences). 100 µl of resin were used per 6 ml of total cell-free reaction. Purification was performed as specified by the manufacturer; all buffers contained 0.1 % DDM and 1mM DTT. Additional washing step with 0.2 % DDM and 300 mM NaCl buffer followed by equilibrating the column back to 0.1% DDM and 150 mM NaCl buffer was added before elution. The final elution buffer contained 100 mM Tris-HCl pH 8, 150 mM NaCl, 1 mM EDTA and 50 mM biotin (BXT buffer, IBA Lifesciences) with addition of 0.1 % DDM and 1 mM DTT. When the pH of the buffer was changed, the sample was additionally dialyzed overnight into buffer containing 100 mM Tris-HCl pH 7.2, 150 mM NaCl, 1 mM EDTA and 0.1% DDM on Slide-A-lyzer device with 3.5 kDa molecular weight cut-off (MWCO) (Thermo Fisher Scientific).

Reconstitution of NS4B into proteoliposomes

Either egg yolk phosphatidylcholine (PC) (L-α-phosphatidylcholine, 99 % pure, Sigma-Aldrich), a mixture (70/30, w/w) of egg yolk PC and cholesterol (Chol) (99 % pure, Sigma-Aldrich) or synthetic lipids 1,2-dimyristoyl-sn-glycero-3-phosphocholine (DMPC) (Avanti Polar Lipids) and 1,2-dipalmitoyl-sn-glycero-3-phosphocholine (DPPC) (Avanti Polar Lipids) were used for lipid reconstitution. Lipids were solubilized using Triton X-100 (Sigma-Aldrich) or DDM with a detergent-to-lipid ratio (DLR) of 10 (mol/mol). Purified full-length NS4B, solubilized in 0.1% DDM, was then mixed with the lipids at a defined lipid-to-protein ratio (w/w). To facilitate the reconstitution step, direct addition of cyclodextrin sugars [3] or dialysis in the presence of Bio-Beads (Bio-Rad) was performed [4].

To reconstitute NS4B into proteoliposomes by the cyclodextrin method, detergent was removed by direct addition of either α- or methyl-β-cyclodextrin in twenty-five steps in linearly increasing amount of cyclodextrin from 0.25 % to 7 % of total amount needed for full reconstitution. The reaction mixture was incubated for 15 min on ice after every step of cyclodextrin addition. When the total amount of cyclodextrin needed for reconstitution was added, reaction mixture was incubated overnight at 4 °C.

The reconstitution by dialysis was performed for 9 days at 4 °C against a buffer containing 100 mM Tris-HCl pH 8, 150 mM NaCl, 1 mM EDTA, 1 mM DTT and hydrophobic polystyrene beads (Bio-Beads SM-2 adsorbents, Bio-Rad) [1]. Protein-lipid-detergent mixture was enclosed in a dialysis membrane with a MWCO of 6-8000 Da (Spectra/Por). A ratio Bio-Beads-to-detergent of 100 (w/w) was used.

Precipitation assay

To establish the minimal amount of cyclodextrin necessary to remove the detergent in the reconstitution reaction, several precipitation assays were performed. Detergent solubilized NS4B protein in presence or absence of detergent solubilized lipids was mixed with various concentrations of α-cyclodextrin or methyl-β-cyclodextrin and incubated for 24 hours at 4 °C followed by 1-hour centrifugation at 25,000 *g*. Full precipitation of the protein was used as a read out for successful detergent removal. Presence of detergent-solubilized NS4B protein in the supernatant or detergent-free, cyclodextrin-precipitated NS4B protein in the pellet was analyzed by SDS-PAGE and Coomassie staining.

Minimal amount of CD necessary to precipitate detergent solubilized NS4B in absence of lipids was determined by incubation of 0.25 nmol of NS4B protein (15.5 µl, 0.5 µg/µl, 16.13 µM) in a buffer containing 0.1 % (w/w) DDM (15.5 µl, 1.96 mM, 30.4 nmol) with increasing concentration of α-cyclodextrin in the range from 30 to 190 nmol. Additionally, 0.25 nmol of NS4B (16.13 µM) in 0.1% DDM (30.4 nmol) was incubated with increasing concentrations of methyl-β-cyclodextrin in the range from 65 to 75 nmol.

Two additional precipitation assays were performed to establish how much cyclodextrin is needed to remove the total amount of detergent from the reaction in the presence of detergent solubilized lipids. First, 0.2 nmol of NS4B (12.4 µl, 0.5 µg/µl, 16.13 µM) in a buffer contacting 0.1% DDM (12.4 µl, 1.96 mM, 24.3 nmol) was mixed with egg yolk phosphatidylcholine lipids with cholesterol in a ratio 70/30 (w/w) (5 µg/µl) solubilized in 3.95% DDM (detergent-to-lipid ratio of 10) at a lipid-to-protein ratio of 2. The protein-lipid-detergent mixture was then incubated with increasing concentration of α-cyclodextrin in the range from 230 to 410 nmol. Moreover, 0.25 nmol of NS4B (15.5 µl, 0.5 µg/µl, 16.13 µM) solubilized in 0.1% DDM (15.5 µl, 1.96 mM, 30.4 nmol) and in the presence of additional 30 nmol of Triton X-100 was incubated with increasing concentration of methyl-β-cyclodextrin in the range from 90 to 120 nmol.

Analysis of reconstituted NS4B by sucrose density-gradient ultracentrifugation

Eighty-five µg of NS4B protein (0.5 µg/µl) in 0.1% DDM buffer was reconstituted into 170 µg of PC/Chol lipids (5 µg/µl) solubilized in 3.95 % DDM or 5 % Triton X-100 (DLR of 10) by direct addition of 5.34 µmol of α-cyclodextrin or 4.79 µmol of methyl-β-cyclodextrin, respectively. The amount of cyclodextrin needed for the reaction was calculated from the precipitation assay described above. Reconstituted NS4B in liposomes was applied on the discontinuous sucrose gradient (5-55%) and centrifuged for 15 h at 200,000 *g*. Twenty-two fractions of the gradient (collected from top to bottom) together with a

pellet were analyzed by SDS PAGE followed by Coomassie-blue staining. The densities of harvested fractions were calculated from refractive index values, measured using a RX-5000α refractometer (Atago).

Preparation of dUL NS4B samples in the presence of ATP used for three-dimensional NMR experiments at 110 kHz

NS4B was expressed in 12 ml of a total reaction mixture using 0.5 ml of wheat germ extract in the presence of 0.1% MNG-3 and 6 mM [^{2}H,^{13}C,^{15}N]-labelled amino acids ("Cell-free" amino acid mix 20 AA, Cambridge Isotope Laboratories, Inc.) for 16 h at 22 °C.

After protein expression, the reaction mixture was incubated with benzonase and 0.25% DDM and centrifuged at 40,000 *g* and 4 °C for 40 min. 200 µl of *Strep*-Tactin resin was used for purification and after washing steps, NS4B was eluted in BXT buffer with 0.1% DDM and 1 mM DTT. The sample was dialyzed overnight against buffer containing 100 mM Tris-HCl pH 7.2, 150 mM NaCl, 1 mM EDTA, 0.1 % DDM and 1 mM DTT.

Purified NS4B protein (0.32 mg) was reconstituted into PC/Chol (70/30) lipids mixture (0.64 mg) at LPR 2 solubilized in Triton X-100 (lipid-to-detergent ratio 1 to 10) by direct stepwise addition of methyl-β-cyclodextrin (46.8 µmol). 2.5-fold excess of cyclodextrin over minimal amount necessary for protein precipitation was used for the reconstitution process. Reconstitution was performed in the presence of 5 mM ATP and 5 mM $MgCl_2$.

Reconstituted proteoliposomes were purified by sedimentation onto a 60% Nycodenz (Alere Technologies) cushion overlaid by 8% Nycodenz in the presence of 5 mM ATP and 5 mM $MgCl_2$ to separate putative free lipids and aggregated protein from proteoliposomes. The final proteoliposomal fraction sedimented on the 60% Nycodenz was harvested and washed three times with buffer containing ATP (100 mM Tris-HCl pH 7.2, 150 mM NaCl, 1 mM EDTA, 1 mM DTT, 5 mM ATP and 5 mM $MgCl_2$) and sedimented into a NMR rotor.

Solid-state MAS NMR experiments

Experiments were performed at a Bruker AVANCE III wide-bore spectrometer with a magnetic field strength of 20 T, corresponding to a ^{1}H Larmor frequency of 850 MHz**.** Two-dimensional [^{1}H,^{15}N]-correlation experiments were recorded at 54 kHz and 60 kHz magic angle spinning frequency using a Bruker 1.3 mm MAS probe, at 80 kHz and 90 kHz MAS using an 0.8 mm MAS probe constructed by Ago

Samoson and co-workers (Tallinn, Estonia) and at 100 kHz and 110 kHz MAS using a Bruker 0.7 mm MAS probe. For experimental details we refer to Table S7. DSS (4,4-dimethyl-4-silapentane-1-sulfonic acid) was used for referencing as internal standard. The sample temperature was 23 °C, as estimated based on the supernatant water line[5].

Bulk ^{1}H and ^{15}N coherence life times (T_2') were measured in a series of 1D experiments by recording the first increment of the [^{1}H,^{15}N]-correlation experiment (see above) and introducing a spin-echo either directly prior to ^{1}H detection for the ^{1}H coherence life time measurement or prior to t_1 evolution for the ^{15}N coherence life time measurement. The spin-echo based experiments were recorded in an interleaved fashion with six or twelve different relaxation delays. ^{15}N and ^{1}H $T_{1\rho}$ times were recorded by replacing the spin-echo block by a spin-lock period, the spin-lock RF amplitude was 13 kHz for the ^{1}H and 13 kHz for the ^{15}N experiment. Otherwise experimental settings were identical to the [^{1}H,^{15}N]-correlation experiment (see Table S7).

CP- and DREAM transfer based 3D correlation experiments for backbone assignment, including hCANH, hCONH and hCAcoNH, on the uniformly labelled dUL NS4B protein were recorded at 20 T, 110 kHz MAS and 23˚C using a Bruker 0.7mm MAS rotor. The hCBcaNH experiment was recorded at 60 kHz using a Bruker 1.3 mm MAS rotor. For experimental details we refer to Table S6.

CP-based hCANH and hCONH spectra on dGVL NS4B protein were recorded using a very similar parameter set (Table S8). Spectra were processed using the Bruker TOPSPIN 3.5 software package and analyzed using the CCPN software [6]. The 2D spectra were processed using a 72° shifted sine bell function (QSINE, SSB=2.5) and plotted just above noise level. Relaxation data were analyzed using the TOPSPIN 3.5. Dynamics module. ^{1}H and ^{15}N linewidth (referred to also as FWHM in the manuscript) measurements were performed using TOPSPIN 3.5: 1D traces were extracted from 2D ^{1}H-^{15}N correlation spectra, after processing with QSINE, SSB 2.5, and fitted using the TOPSPIN 3.5 Line Shapes module.

Supporting Results

Determination of micelle sizes

The number of detergent molecules bound to NS4B (DDM^{NS4B}), as well as the α-cyclodextrin/DDM ratio ($R^{\alpha\mathrm{CD/DDM}}$) can be deduced from the two coupled equations describing the precipitation reactions, with αCD^{total} being the amount of α-cyclodextrin needed for precipitation:

$$R^{\alpha\mathrm{CD/DDM}} \times \left(DDM^{micelle} + DDM^{NS4B}\right) = \alpha CD^{total}$$
$$R^{\alpha\mathrm{CD/DDM}} \times (120 + DDM^{NS4B}) = 440$$

and

$$R^{\alpha\mathrm{CD/DDM}} \times \left(DDM^{micelle} + DDM^{NS4B} + DDM^{lipid}\right) = \alpha CD^{total}$$
$$R^{\alpha\mathrm{CD/DDM}} \times (120 + DDM^{NS4B} + 950) = 1950$$

This results in DDM^{NS4B} equal 160 (in nmol), and $R^{\alpha\mathrm{C\ D/DDM}}$ equal 1.6.

A similar calculation results for mβ-CD:

$$R^{\mathrm{m}\beta\mathrm{CD/DDM}} \times \left(DDM^{micelle} + DDM^{NS4B}\right) = m\beta CD^{total}$$
$$R^{\mathrm{m}\beta\mathrm{CD/DDM}} \times (120 + DDM^{NS4B}) = 300$$

results in $R^{\mathrm{m}\beta\mathrm{CD/DDM}}$ equal 1.1 (with DDM^{NS4B} equal 160),

and

$$R^{\mathrm{m}\beta\mathrm{CD/DDM}} \times \left(DDM^{micelle} + DDM^{NS4B}\right) + \left(R^{\mathrm{m}\beta\mathrm{CD/TX100}} \times TX100_{lipid}\right) = m\beta CD^{total}$$
$$1.1 \times (120 + 160) + \left(R^{\mathrm{m}\beta\mathrm{CD/TX100}} \times 950\right) = 1730.$$

results in $R^{\mathrm{m}\beta\mathrm{CD/TX100}}$=1.5.

Supporting Figures

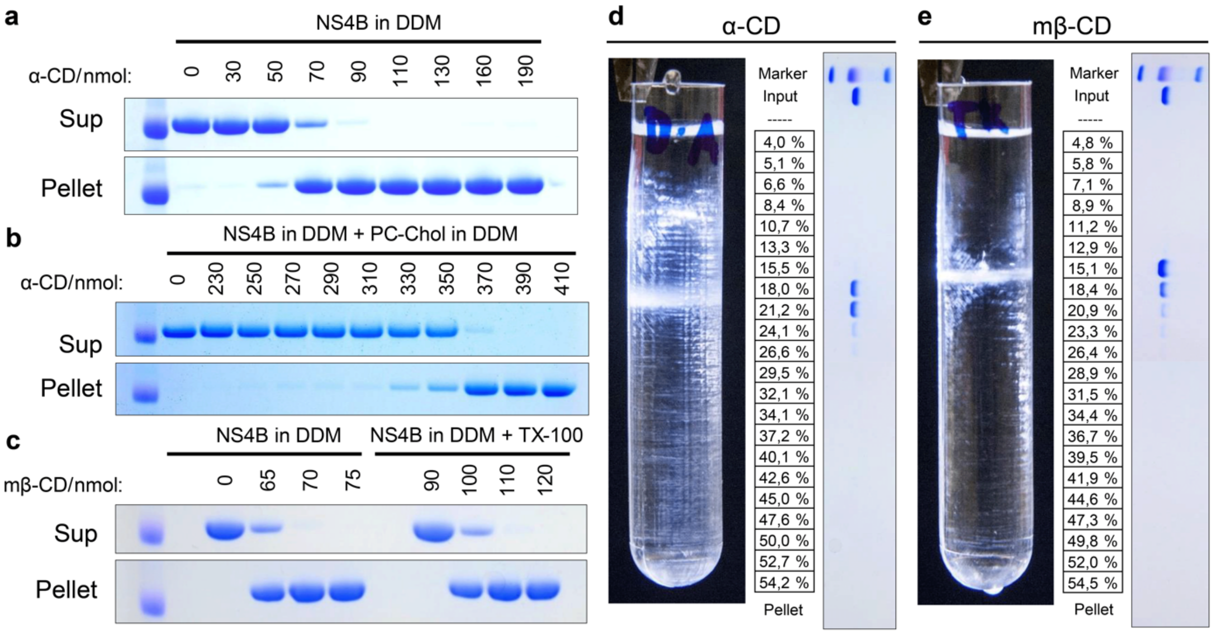

Figure S1. Cyclodextrin mediated lipid reconstitution of NS4B yields a homogenous proteoliposome sample. Amount of α-CD necessary to precipitate (a) 0.25 nmol of NS4B in 0.1% DDM, and (b) 0.2 nmol of NS4B in 0.1% DDM mixed with PC/Chol at LPR 2 solubilized in DDM. (c) Amount of mβ-CD necessary to precipitate 0.25 nmol of NS4B in 0.1% DDM alone or in the presence of additional 30 nmol Triton X-100. (d-e) Migration of NS4B proteoliposomes in a sucrose gradient. (d) 85 µg of DDM-solubilized NS4B protein was reconstituted using 170 µg of PC/Chol at LPR 2 by the gradual addition of 5.34 µmol of α-CD for lipids solubilized in DDM; (e) 4.79 µmol of mβ-CD for lipids solubilized in Triton X-100. Tables with percentage values represent sucrose concentration of harvested gradient fractions analyzed by SDS PAGE Coomassie on the right.

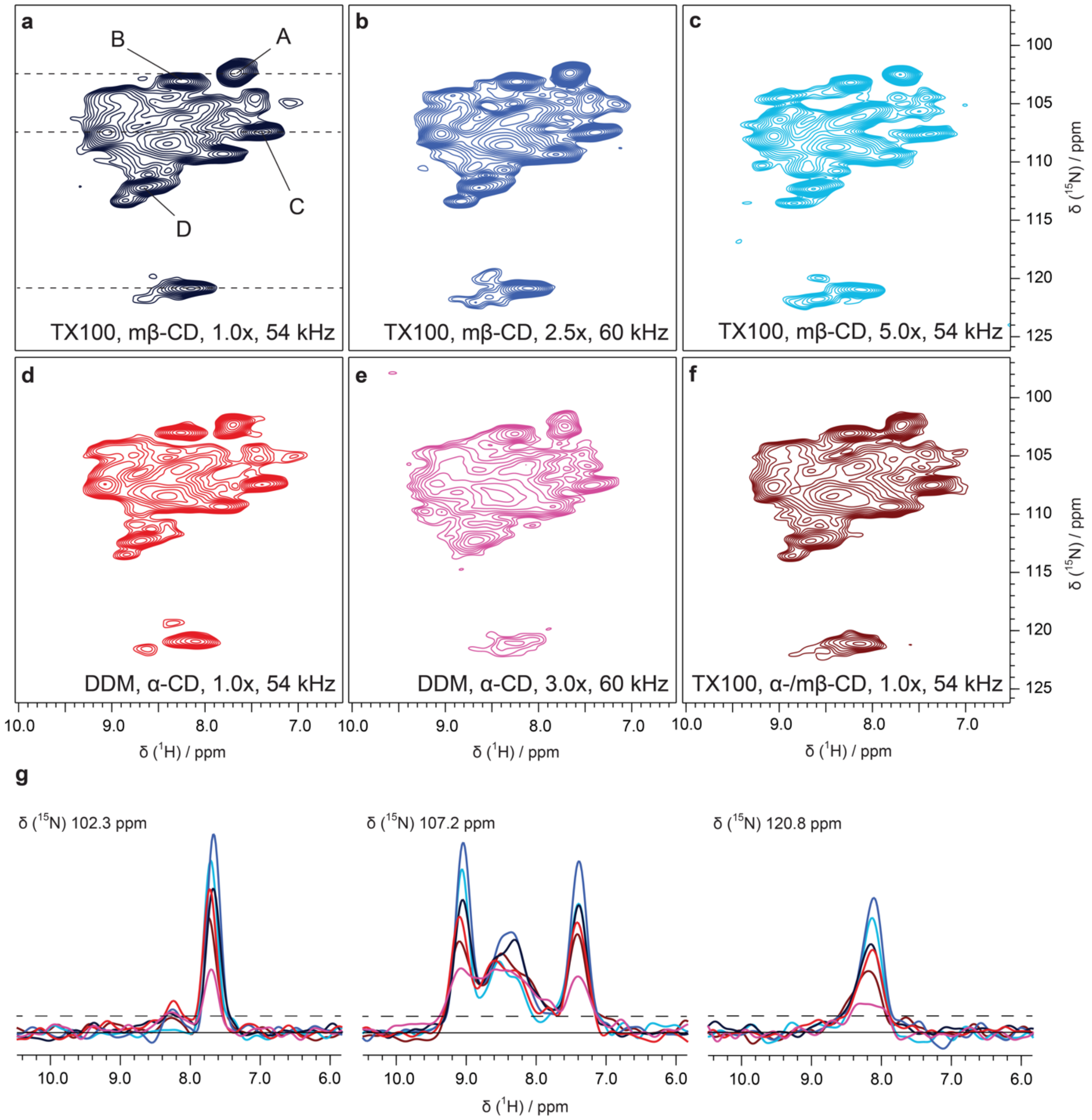

Figure S2: 2D ^{1}H-^{15}N spectra of NS4B as a function of proteoliposome reconstitution using different detergent/cyclodextrin combinations and concentrations. (See also Table S2.) DDM solubilized NS4B protein was reconstituted into egg yolk PC/Chol liposomes at LPR 2. Input lipids were solubilized in Triton X-100 (a-c, f) or in DDM (d, e). Detergent molecules were removed during the reconstitution process by methyl-β-cyclodextrin (a-c), α-cyclodextrin (d, e) or by their combination (f), with the minimum amount necessary to remove the detergent (a, d, f) defined by the precipitation assay, or by 2.5 fold (b), 5 fold (c), and 3 fold (e) excess over the minimum amount necessary to remove the detergent. The ^{1}H-^{15}N correlation spectra were acquired at 23°C for 16 hours with 256 scans at 54 kHz or 60 kHz MAS. The 2D spectra were processed using a 72° shifted sine bell function (QSINE, SSB=2.5) and plotted just above noise level. (g) Selected 1D traces show extracts of the 2D plot of samples with different reconstitution conditions. Dashed line in (g) represent noise level cut off.

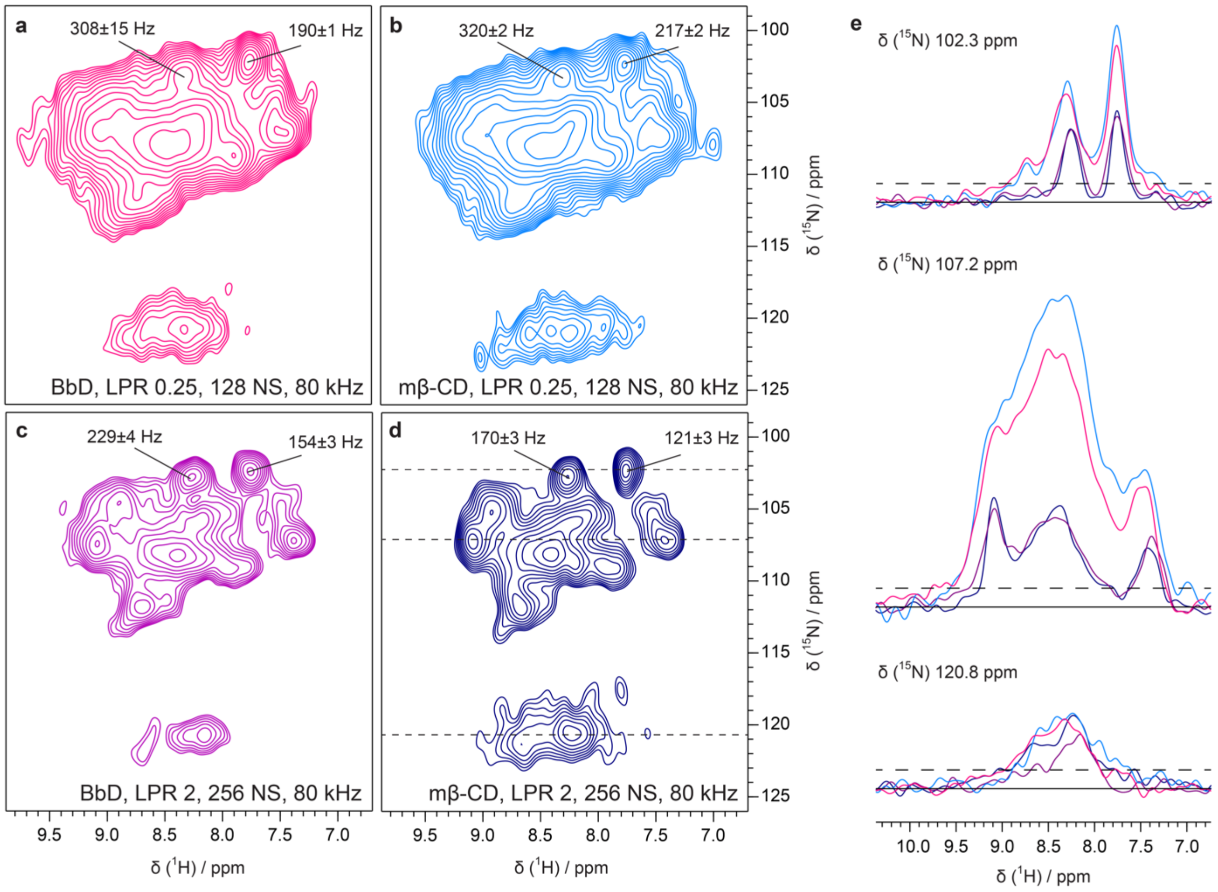

Figure S3: NS4B reconstituted by dialysis or by addition of mβ-cyclodextrin yields spectra of similar SNR and resolution. dGY NS4B was reconstituted into PC/Chol liposomes with a LPR 0.25 and c LPR 2 by detergent removal with dialysis in the presence of Bio-Beads (BbD) or with stepwise direct addition of mβ-cyclodextrin (mβ-CD) and b LPR 0.25 and d LPR 2. Samples were sedimented into a 0.8 mm rotor and ^{1}H-^{15}N correlation spectra were acquired at 80 kHz MAS at 23°C for 4 hours with 128 scans (NS) or 8 hours with 256 scans. The 2D spectra were processed using a 72° shifted sine bell function (QSINE, SSB=2.5) and plotted just above noise level. Dashed lines represent position of 1D traces in (e). e Selected 1D traces show extracts of the 2D plot of Bio-Beads reconstituted sample for LPR 0.25 (pink) and LPR 2 (violet) and cyclodextrin reconstituted sample with LPR 0.25 (light blue) and LPR 2 (dark blue). Dashed line in (e) represent noise level cut off.

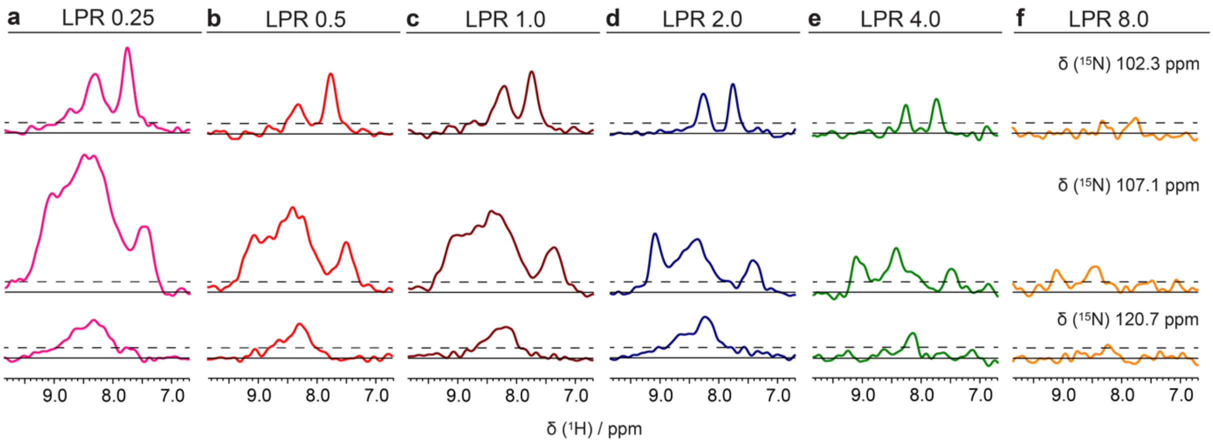

Figure S4. **Spectral resolution of 2D ^{1}H-^{15}N spectra as a function of LPR**. Representative 1D traces of the two-dimensional ^{1}H-^{15}N correlation spectra of dGY NS4B reconstituted into PC/Chol liposomes at LPRs from 0.25 to 8 (Figure 2). The dashed lines represent the noise level cut off applied in the 2D spectra. Lipid reconstitution was achieved by Bio-Beads-enhanced dialysis (a-c) or using mβ-CD (d-f). Full width at half maximum (FWHM) and SNR are given in Table S3

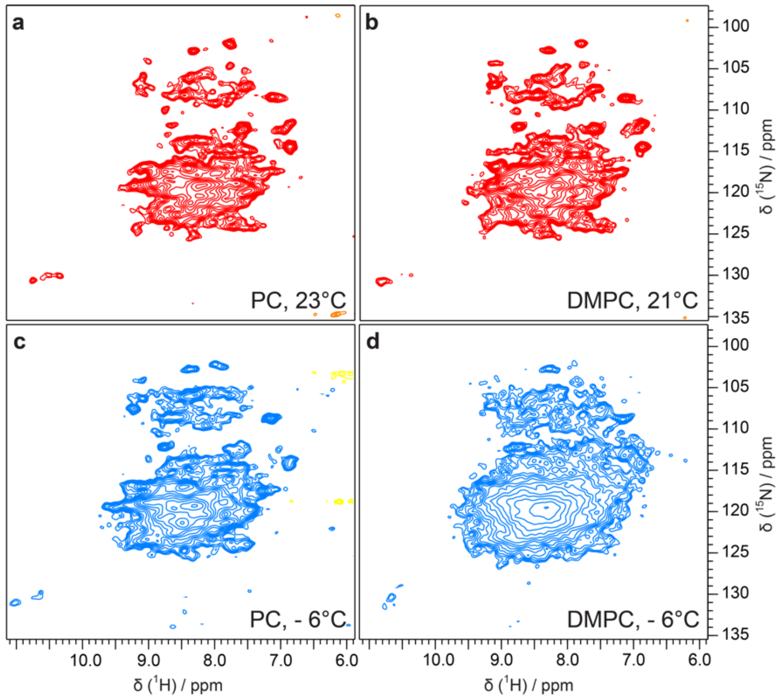

Figure S5: 2D ^{1}H-^{15}N spectral resolution correlates with the lipid transition temperature. dUL NS4B was reconstituted at LPR 2 in PC lipids a, c or DMPC lipids b, d and sedimented into a 0.7 mm rotor. All [^{1}H,^{15}N] correlation spectra were acquired for 2 hours with 32 scans at 60 kHz MAS at room temperature a, b or at a temperature below zero c, d. Numerical values of the homogeneous and the incoherent contribution to the linewidth, as extracted from bulk relaxation measurements, are given in Table S4.

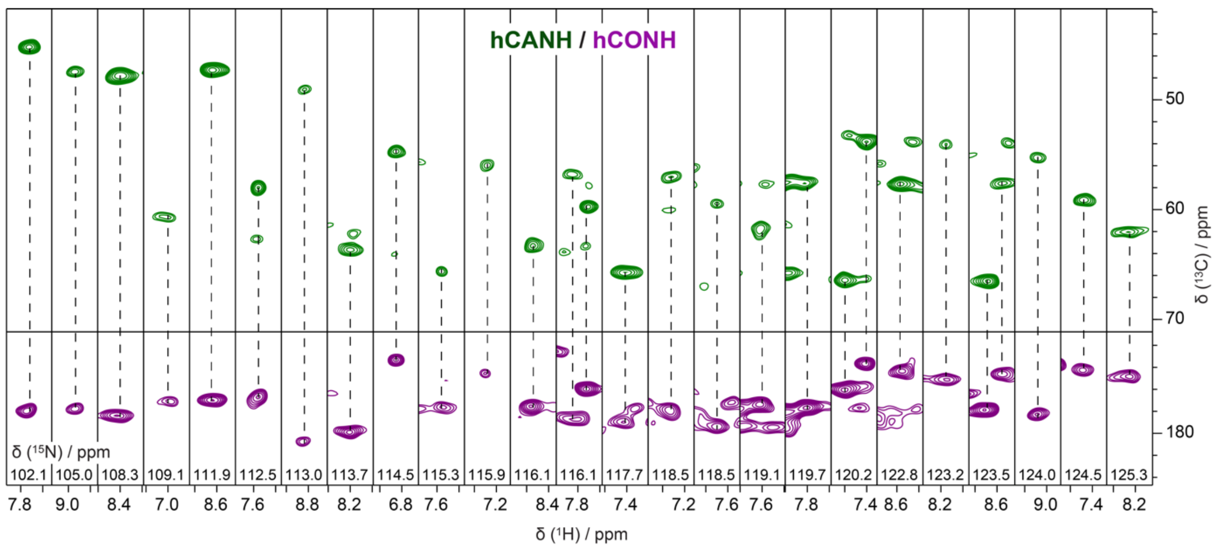

Figure S6: Strips connecting resonances in the 3D hCONH and hCANH correlation spectra. dUL NS4B was reconstituted into PC/Chol lipids at LPR 2 in the presence of ATP. Green contours denote peaks from the 3D hCANH spectrum, violet contours the ones from the hCONH.

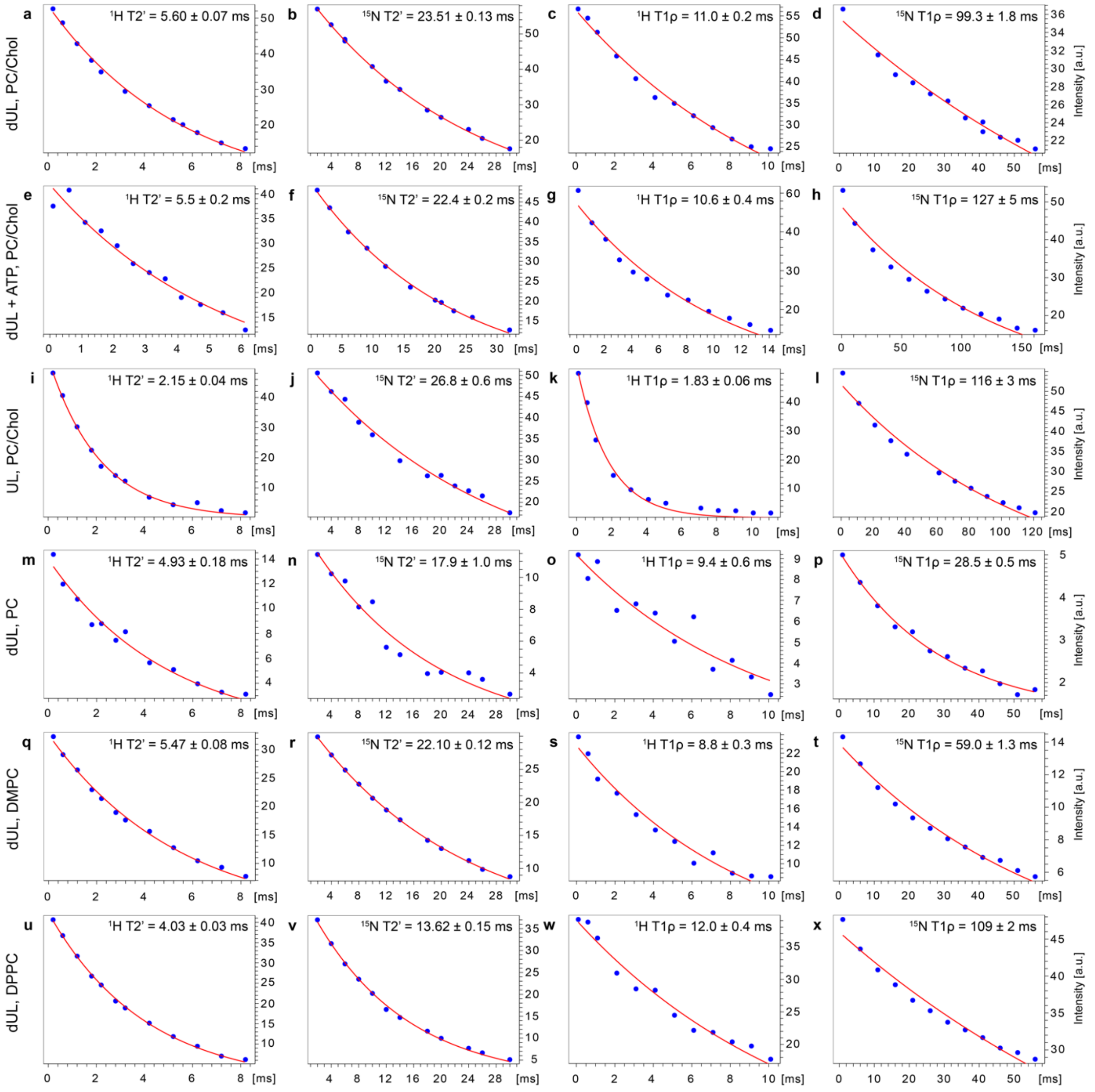

Figure S7: Mono-exponential fit of ^{1}H and ^{15}N T_2' and $T_{1\rho}$ data in sample condition screening (Summarized in Table 1).

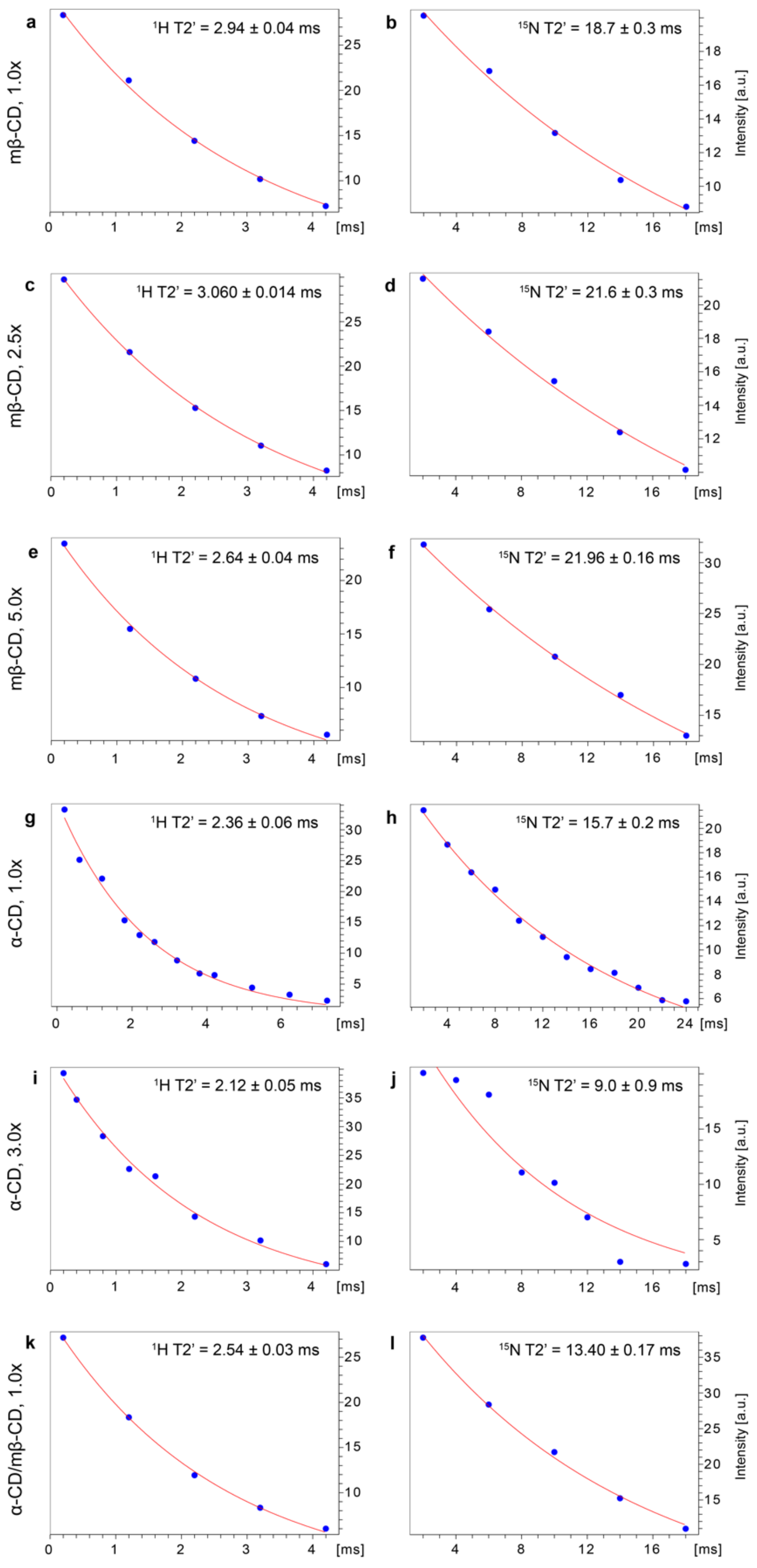

Figure S8: Mono-exponential fit of ^{1}H and ^{15}N T_2' data in cyclodextrin condition screening (Summarized in Table S2).

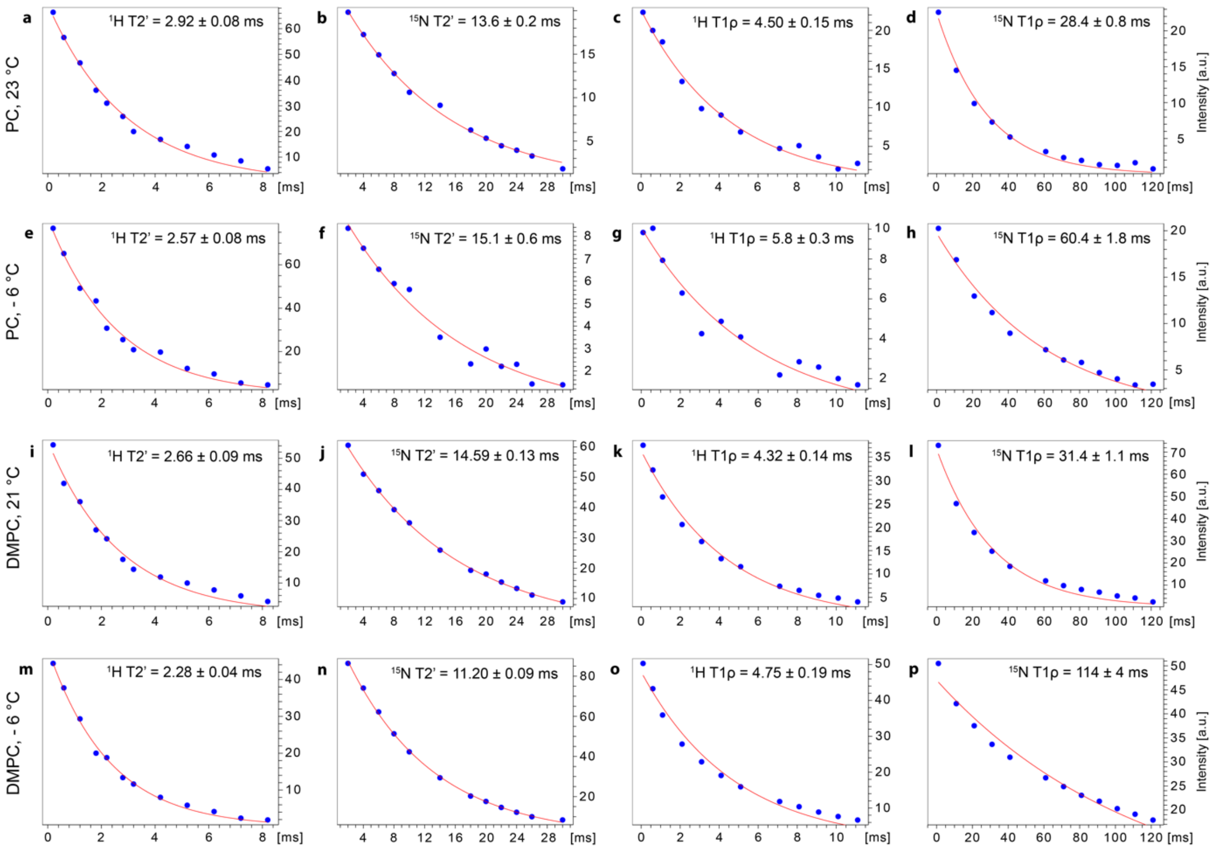

Figure S9: Mono-exponential fit of ^{1}H and ^{15}N T_2' and $T_{1\rho}$ data in temperature screening experiment (Summarized in Table S4).

Supporting Tables

	α-cyclodextrin precipitation				methyl-β-cyclodextrin precipitation				
	NS4B in 0.1% DDM		plus PC/Chol in DDM		NS4B in 0.1% DDM		plus additional TX-100		plus PC/Chol in TX100
	Exp. Values	Norm. Values	Exp. Values	Norm. Values	Exp. Values	Norm. Values	Exp. Values	Norm. Values	Norm. Values
Reaction volume (µl)	15.5		12.4		15.5		15.5		
**NS4B**									
Molar amount (nmol)	0.25	1	0.2	1	0.25	1	0.25	1	1
Mass (µg)	7.77	31.06	6.2	31.06	7.77	31.06	7.77	31.06	31.06
Molar concentraton (µM)	16.3	16.3	16.3	16.3	16.3	16.3	16.3	16.3	16.3
Weigth concentration (µg/µl)	0.5	0.5	0.5	0.5	0.5	0.5	0.5	0.5	0.5
**"Free" DDM in a buffer**									
Molar amount (nmol)	30.4	121.6	24.3	121.5	30.4	121.6	30.4	121.6	121.6
Molar concentraton (mM)	1.96	1.96	1.96	1.96	1.96	1.96	1.96	1.96	1.96
Weigth concentration (mg/µl)	0.1	0.1	0.1	0.1	0.1	0.1	0.1	0.1	0.1
**Additional detergent**									
Amount of detergent (nmol)	-	-	190	950	-	-	30	120	950
**Cyclodextrin**									
**α-cyclodextrin (nmol)**									
- total amount	110	440	390	1950					
- bound to "A" and "B" fractions	110	440	n.d.	440					
- bound to "C" fraction	n.a.	n.a.	n.d.	1510					
**methyl-β-cyclodextrin (nmol)**									
- total amount					75	300	120	480	1730
- bound to "A" and "B" fractions					75	300	n.d.	300	300
- bound to "D" fraction					n.a.	n.a.	n.d.	180	1430
**Cyclodextrin/detergent ratio**									
α-CD / DDM				**1.6**					
mβ-CD / TX-100								**1.5**	
mβ-CD / DDM						**1.1**			
**DDM and CD amounts (nmol) - back calculated from CD/DDM ratio**									
DDM in "A" + "B" fractions		277				277			
Unbound DDM - fraction "A"		122				122			
CD bound to "A" fraction of DDM		193				132			
Protein bound DDM - fraction "B"		155				155			
CD bound to "B" fraction of DDM		247				168			
**Amounts of cyclodextrin (µmol) for reconstitution of 1 nmol of NS4B in 0.1% DDM into PC/Chol at LPR 2 (lipid solubilized at lipid-detergent ratio 10)**									
α-CD binding "A" + "B" fractions		0.44							
α-CD binding "C" fraction		1.51							
**Total amount of α-cyclodextrin**		**1.95**							
mβ-CD binding "A" + "B" fractions		0.30							
mβ-CD binding "D" fraction		1.43							
**Total amount of mβ-cyclodextrin**		**1.73**							

Table S1: Experimental parameters of cyclodextrin precipitation assay and NS4B lipid reconstitution. "A" represent fraction of unbound DDM in a buffer. "B" represent fraction of DDM bound to protein and "C" and "D" represent DDM and Triton X-100 used for lipid solubilization (for graphical representation see Figure 1a, b).

Cyclodextrin [a]	mβ-CD	mβ-CD	mβ-CD	α-CD	α-CD	mβ-CD/α-CD
**Detergent** [b]	**TX-100**	**TX-100**	**TX-100**	**DDM**	**DDM**	**TX-100**
**Recon factor** [c]	**1.0x**	**2.5x**	**5.0x**	**1.0x**	**3.0x**	**1.0x**
**MAS (kHz)**	54	60	54	54	60	54
**^{1}H R2'/π (Hz)**	108 ± 2	104 ± 1	121 ± 2	135 ± 3	150 ± 4	125 ± 2
**^{15}N R2'/π (Hz)**	17 ± 1	15 ± 1	15 ± 1	20 ± 1	36 ± 4	24 ± 1
**FWHM (Hz)**						
**peak A**	170 ± 7	149 ± 9	157 ± 8	156 ± 7	175 ± 9	168 ± 8
**peak B**	216 ± 8	191 ± 7	177 ± 7	250 ± 6	259 ± 11	257 ± 6
**peak C**	226 ± 9	211 ± 6	239 ± 5	250 ± 11	351 ± 15	217 ± 9
**peak D**	233 ± 7	215 ± 9	189 ± 6	229 ± 9	322 ± 8	245 ± 10
**SNR**						
**peak A**	19.3	26.8	20.8	17.9	8.1	15.9
**peak B**	19.7	26.9	20.0	14.9	9.3	15.2
**peak C**	16.6	21.4	15.8	14.6	7.9	13.6
**peak D**	16.0	18.9	17.6	12.0	6.9	11.4

[a] cyclodextrin used for protein reconstitution
[b] detergent used for lipid solubilization
[c] multiplicity of minimal amount for detergent removal by cyclodextrin

Table S2: Bulk ^{1}H and ^{15}N R2‘ rate constants of amide groups of NS4B proteoliposomes, reconstituted using different detergents and cyclodextrins compositions. The related spectra are shown in Figure S2. Data were recorded at 850 MHz and 54 kHz and 60 kHz MAS. The total experimental time was 16 h for each spectrum (see Table S7, column 1 for experimental details). Experimental raw data (spectral intensities as a function of the relaxation delay) and the fitted intensity decay curves are shown in Figure S8.

	LPR 0.25	LPR 0.5	LPR 1.0	LPR 2.0	LPR 4.0	LPR 8.0
**Reconstitution**	Dialysis	Dialysis	Dialysis	mβ-CD	mβ-CD	mβ-CD
**Number of scans**	128	128	128	256	400	480
**Time**	4h 7m	4h 14m	4h 14m	8h 15m	13h 13m	15h 52m
**Protein content (%)** [a]	80%	66%	50%	33%	20%	11%
**FWHM (Hz)**						
**peak A**	308 ± 15	264±5	262±3	170±3	130±4	-
**peak B**	190 ± 1	174±1	169±3	121±3	116±3	-
**SNR** [b]						
**peak A**	23	12	14	11**	4**	-
**peak B**	19	11	11	9**	3**	-

[a] percentage of protein amount in proteoliposome dry mass
[b] The SNR has been scaled relative to the first spectrum (LPR 0.25) by the square-root of the ratio of the recording times.

Table S3. Determination of the optimal LPR and cyclodextrin condition. Experimental details of dGY NS4B spectra at LPRs from 0.25 to 8 that are shown in Figure 2.

Lipids	hNH	T (°C)	Tm (°C)	^{1}H		^{15}N	
				R2'/π (Hz)	R1ρ/π (Hz)	R2'/π (Hz)	R1ρ/π (Hz)
PC	Fig. S5a	23	-6	109 ± 3	71 ± 2	24 ± 1	11 ± 1
PC	Fig. S5c	-6	-6	124 ± 4	55 ± 3	21 ± 1	5 ± 1
DMPC	Fig. S5b	21	24	120 ± 4	74 ± 2	22 ± 1	10 ± 1
DMPC	Fig. S5d	-6	24	140 ± 3	67 ± 3	28 ± 1	3 ± 1

Table S4. Temperature-dependent bulk homogeneous linewidths R2'/π and R1ρ/π measured for NS4B in different lipid environment (2D spectra are shown in Figure S5). Data were recorded at 60 kHz MAS using a Bruker 0.7 mm MAS rotor. Experimental raw data (spectral intensities as a function of the relaxation delay) and the fitted intensity decay curves are shown in Figure S9.

FWHM and SNR of individual peaks:

dUL, PC/Chol

Sample 1

	FWHM			SNR
**A**	102	±	7	4.4
**B**	114	±	5	8.6
**C**	120	±	7	5.4
**D**	101	±	13	6.0
**E**	113	±	3	10.7
**F**	124	±	6	6.3
**G**	130	±	8	8.0
**H**	102	±	5	6.5
**I**	105	±	18	10.6
**J**	129	±	6	6.2

Sample 2

	FWHM			SNR
**A**	106	±	9	4.6
**B**	115	±	6	6.1
**C**	144	±	14	4.2
**D**	167	±	8	6.0
**E**	117	±	3	10.1
**F**	129	±	8	4.7
**G**	132	±	5	7.5
**H**	104	±	3	8.0
**I**	163	±	9	12.0
**J**	126	±	16	6.1

dUL + ATP, PC/Chol

Sample 1

	FWHM			SNR
**A**	101	±	7	9.4
**B**	108	±	5	9.5
**C**	175	±	7	5.8
**D**	112	±	13	12.6
**E**	90	±	3	6.6
**F**	145	±	6	10.1
**G**	98	±	8	8.4
**H**	164	±	5	14.6
**I**	117	±	18	9.0

Sample 2

	FWHM			SNR
**A**	99	±	9	7.5
**B**	109	±	6	9.1
**C**	134	±	14	6.1
**D**	102	±	8	12.5
**E**	129	±	3	6.4
**F**	128	±	8	11.0
**G**	89	±	5	6.8
**H**	123	±	3	15.0
**I**	144	±	9	8.1

UL, PC/Chol

	FWHM			SNR
**A**	146	±	9	3.2
**B**	145	±	8	3.1
**C**	112	±	17	3.8
**D**	84	±	16	3.5
**E**	175	±	8	4.9
**F**	131	±	5	7.0
**G**	193	±	7	5.4
**H**	158	±	8	4.6
**I**	150	±	20	4.9
**J**	130	±	20	4.4

dUL, PCegg

	FWHM			SNR
**A**	86	±	4	5.2
**B**	77	±	4	6.2
**C**	111	±	3	6.0
**D**	101	±	4	11.7
**E**	92	±	5	6.6
**F**	97	±	4	9.0
**G**	73	±	4	7.5
**H**	120	±	50	9.0
**I**	127	±	7	5.9

dUL, DMPC

	FWHM			SNR
**A**	107	±	6	7.3
**B**	81	±	4	11.3
**C**	89	±	5	8.2
**D**	116	±	5	9.4
**E**	81	±	3	14.0
**F**	81	±	6	12.3
**G**	101	±	3	14.0
**H**	87	±	3	12.1
**I**	168	±	14	11.1
**J**	165	±	15	7.0

Average FWHM and SNR values:

dUL, PC/Chol

	FWHM			SNR
**A-J**	120	±	20	7 ± 2

dUL + ATP, PC/Chol

	FWHM			SNR
**A-I**	120	±	30	9 ± 3

UL, PC/Chol

	FWHM			SNR
**A-J**	140	±	30	4.5±1.2

dUL, PCegg

	FWHM			SNR
**A-I**	100	±	20	8 ± 2

dUL, DMPC

	FWHM			SNR
**A-J**	110	±	30	11 ± 3

Table S5: Individual linewidths of isolated peaks in the 2D spectra of NS4B in different lipid environments shown in Figure 3 used for calculation of average FWHM in Table 1.

	**hCANH**	**hCONH**	**hCAcoNH**	**hCBcaNH**
Labelling scheme	dUL	dUL	dUL	dUL
MAS frequency / kHz	110	110	110	60
Field / T	20	20	20	20
Interscan recovery delay / s	1.7	1.7	1.7	1.8
Number of scans	8	8	96	32
Total experimental time /d	3.3	3.4	8.1	5.1
**1st transfer**	**H-CA CP**	**H-CO CP**	**H-CA CP**	**H-CB CP**
^{1}H field/ kHz	136	137	139	96
^{13}C field / kHz	34	33	42	43
Shape	Tangent_45-55 ^{1}H	Tangent_45-55 ^{1}H	Tangent_45-55 ^{1}H	Tangent_45-55_80 ^{1}H
Time / ms	4.4	3.8	7.6	5.75
**DREAM transfer**	**-**	**-**	**CA-CO**	**CB-CA**
^{13}C carrier / ppm	-	-	88	30
^{13}C field / kHz	-	-	52	31
Shape	-	-	Tangent_45-55_80 ^{13}C	Tangent_45-55_80 ^{13}C
Time / ms	-	-	13	4
**2nd transfer**	**CA-N CP**	**CO-N CP**	**CO-N CP**	**CA-N CP**
^{13}C field / kHz	65	66	67	36
^{15}N field / kHz	39	38	42	20
Shape	Tangent_45-55 ^{13}C	Tangent_45-55 ^{13}C	Tangent_45-55 ^{13}C	Tangent_45-55_80 ^{13}C
Time / ms	20	18	13	10
**3rd transfer**	**N-H CP**	**N-H CP**	**N-H CP**	**N-H CP**
^{15}N field / kHz	20	20	20	39
^{1}H field/ kHz	83	84	77	94
Shape	Tangent_60-40_80 ^{1}H	Tangent_60-40_80 ^{1}H	Tangent_60-40_80 ^{1}H	Tangent_60-40_80 ^{1}H
Time / ms	0.7	0.75	0.7	1.1
**direct dimension (^{1}H)**				
^{1}H carrier / ppm	0	0	0	0
Data points	4096	4096	4096	4096
Spectral width / ppm	47	47	47	47
^{15}N decoupling	WALTZ[1]64	WALTZ64	WALTZ64	WALTZ64
^{15}N decoupling field / kHz	10	10	10	5
^{15}N decoupling pulse length / µs	51.5	51.5	51.5	51.5
Acquisition time / ms	51.6	51.6	51.6	51.6
**indirect dimension (^{13}C)**				
^{13}C carrier / ppm	55	175	55	42
Data points	156	160	52	70
Spectral width / ppm	78	78	39	78
^{1}H decoupling	swf-TPPM[2]	swf-TPPM	swf-TPPM	swf-TPPM
^{1}H decoupling field / kHz	10	10	10	10
^{1}H decoupling pulse length / µs	47	47	47	47
Acquisition time / ms	4.7	4.8	3.1	2.1
**indirect dimension (^{15}N)**				
^{15}N carrier / ppm	117.5	117.5	117.5	117.5
Data points	128	128	72	96
Spectral width / ppm	40	40	40	40
^{1}H decoupling	swf-TPPM	swf-TPPM	swf-TPPM	swf-TPPM
^{1}H decoupling field / kHz	10	10	10	10
^{1}H decoupling pulse length / µs	47	47	47	47
Solvent suppression	MISSISSIPPI[3]	MISSISSIPPI	MISSISSIPPI	MISSISSIPPI
^{1}H suppression field / kHz	20	20	20	10
Length of suppression period / ms	60	60	200	200
Acquisition time / ms	18.6	18.6	10.4	13.9

1. Shaka AJ, Keeler J, Frenkiel T, Freeman R. An improved sequence for broadband decoupling: WALTZ-16. Journal of Magnetic Resonance (1969). Academic Press; 1983 Apr 1;52(2):335–8.
2. Vinod Chandran C, Madhu PK, Kurur ND, Bräuniger T. Swept-frequency two-pulse phase modulation (SWf-TPPM) sequences with linear sweep profile for heteronuclear decoupling in solid-state NMR. Magn Reson Chem. 2008 Oct;46(10):943–7.
3. Zhou DH, Rienstra CM. High-performance solvent suppression for proton detected solid-state NMR. Journal of Biomolecular NMR. 2008 May;192(1):167–72.

Table S6: Experimental parameters of the proton detected 3D experiments, recorded on uniformly labelled NS4B protein reconstituted into PC/Chol at LPR 2 in presence of ATP.

**MAS frequency**	**60 kHz**	**80 kHz**	**110 kHz**
Labelling scheme	dGY	dGY	dUL
Rotor diameter	1.3 mm	0.8 mm	0.7 mm
Field / T	20	20	20
Interscan recovery delay / s	1.5	1.0	1.5
Number of scans	256	256	32
Total experimental time / h	16	8	2
**1st transfer**	**H-N CP**	**H-N CP**	**H-N CP**
^1H field/ kHz	100	64	83
^{15}N field / kHz	41	15	18
Shape	Tangent_40-60_80 ^1H	Tangent_40-60_80 ^1H	Tangent_40-60_80 ^1H
Time / ms	1.2	1.0	1.3
**2nd transfer**	**N-H CP**	**N-H CP**	**N-H CP**
^{15}N field / kHz	46	15	18
^1H field/ kHz	100	64	81
Shape	Tangent_60-40_80 ^1H	Tangent_60-40_80 ^1H	Tangent_60-40_80 ^1H
Time / ms	1.1	1.0	1.3
**direct dimension (^1H)**			
^1H carrier / ppm	2.5	4.8	0
Data points	4096	5550	2048
Spectral width / ppm	79	47	40
^{15}N decoupling	WALTZ16	WALTZ64	WALTZ64
^{15}N decoupling field / kHz	5	5	10
^{15}N decoupling pulse length / µs	50	51.5	26
^{13}C decoupling	-	-	WALTZ16
^{13}C decoupling field / kHz	-	-	5
^{13}C decoupling pulse length / µs	-	-	50
Acquisition time / ms	30	70	30
**indirect dimension (^{15}N)**			
^{15}N carrier / ppm	115	115	116
Data points	128	100	128
Spectral width / ppm	39	100	39
^1H decoupling	WALTZ64	swf-TPPM	swf-TPPM
^1H decoupling field / kHz	10	3.5	10
^1H decoupling pulse length / µs	25	47	46
Solvent suppression	MISSISSIPPI	MISSISSIPPI	MISSISSIPPI
^1H suppression field / kHz	25	5	20
Length of suppression period / ms	50	90	200
Acquisition time / ms	19.2	5.8	19.2

Table S7: Experimental parameters of the proton detected 2D experiments with selectively labelled and uniformly labelled NS4B protein.

	hCANH	hCONH
Labelling scheme	dGVL	dGVL
MAS frequency / kHz	100	100
Field / T	20	20
Interscan recovery delay / s	1.7	1.7
Number of scans	32	32
Total experimental time /d	3	2.8
**1st transfer**	**H-CA CP**	**H-CO CP**
^{1}H field/ kHz	129	124
^{13}C field / kHz	34	33
Shape	Tangent_45-55 ^{1}H	Tangent_45-55 ^{1}H
Time / ms	3.5	3.8
**2nd transfer**	**CA-N CP**	**CO-N CP**
^{13}C field / kHz	63	58
^{15}N field / kHz	38	38
Shape	Tangent_45-55 ^{13}C	Tangent_45-55 ^{13}C
Time / ms	15	17
**3rd transfer**	**N-H CP**	**N-H CP**
^{15}N field / kHz	20	20
^{1}H field/ kHz	74	75
Shape	Tangent_60-40_80 ^{1}H	Tangent_60-40_80 ^{1}H
Time / ms	0.7	0.75
**direct dimension (^{1}H)**		
^{1}H carrier / ppm	0	0
Data points	4096	4096
Spectral width / ppm	47	47
^{15}N decoupling	WALTZ64	WALTZ64
^{15}N decoupling field / kHz	10	10
^{15}N decoupling pulse length / µs	51.5	51.5
Acquisition time / ms	51.6	51.6
**indirect dimension (^{13}C)**		
^{13}C carrier / ppm	55	175
Data points	58	58
Spectral width / ppm	78	78
^{1}H decoupling	swf-TPPM	swf-TPPM
^{1}H decoupling field / kHz	10	10
^{1}H decoupling pulse length / µs	47	47
Acquisition time / ms	4.7	4.8
**indirect dimension (^{15}N)**		
^{15}N carrier / ppm	117.5	117.5
Data points	70	70
Spectral width / ppm	40	40
^{1}H decoupling	swf-TPPM	swf-TPPM
^{1}H decoupling field / kHz	10	10
^{1}H decoupling pulse length / µs	47	47
Solvent suppression	MISSISSIPPI	MISSISSIPPI
^{1}H suppression field / kHz	20	20
Length of suppression period / ms	60	60
Acquisition time / ms	18.6	18.6

Table S8: Experimental parameters of the proton detected 3D experiments, recorded on dGVL NS4B protein reconstituted into PC/Chol at LPR 2 in presence of ATP.

References

[1] M. L. Fogeron, V. Jirasko, S. Penzel, D. Paul, R. Montserret, C. Danis, D. Lacabanne, A. Badillo, J. Gouttenoire, D. Moradpour, R. Bartenschlager, F. Penin, B. H. Meier, A. Bockmann, *J. Biomol. NMR* **2016**, *65*, 87-98.

[2] aK. Takai, T. Sawasaki, Y. Endo, *Nat. Protoc.* **2010**, *5*, 227-238; bM. L. Fogeron, A. Badillo, V. Jirasko, J. Gouttenoire, D. Paul, L. Lancien, D. Moradpour, R. Bartenschlager, B. H. Meier, F. Penin, A. Bockmann, *Protein Expr. Purif.* **2015**, *105*, 39-46.

[3] W. J. Degrip, J. Vanoostrum, P. H. Bovee-Geurts, *Biochem. J.* **1998**, *330* 667-674.

[4] J. L. Rigaud, B. Pitard, D. Levy, *Biochim. Biophys. Acta* **1995**, *1231*, 223-246.

[5] A. Bockmann, C. Gardiennet, R. Verel, A. Hunkeler, A. Loquet, G. Pintacuda, L. Emsley, B. H. Meier, A. Lesage, *J Biomol. NMR* **2009**, *45*, 319-327.

[6] W. F. Vranken, W. Boucher, T. J. Stevens, R. H. Fogh, A. Pajon, M. Llinas, E. L. Ulrich, J. L. Markley, J. Ionides, E. D. Laue, *Proteins* **2005**, *59*, 687-696.
